# Molecular detection and genetic diversity of *Anaplasma* in ticks from southeastern and central Shanxi, China

**DOI:** 10.3389/fmicb.2026.1778059

**Published:** 2026-03-10

**Authors:** Jia Cui, Dan Li, Fengping Wang, Huaxiang Rao, Hongbing Cheng, Liqing Wang, Juan Yu

**Affiliations:** 1 School of Basic Medical Sciences, Changzhi Medical College, Changzhi, China; 2 School of Public Health, Changzhi Medical College, Changzhi, China; 3 Key Laboratory of Cellular Physiology, Ministry of Education, Department of Physiology, Shanxi Medical University, Taiyuan, China

**Keywords:** *16S rRNA*, *Anaplasma*, China, ixodid ticks, phylogeographic analysis

## Abstract

*Anaplasma* is an obligate intracellular gram-negative bacterium belonging to the family Anaplasmataceae and the order Rickettsiales, which is primarily transmitted by the bite of ixodid ticks. To investigate the prevalence risk and genetic diversity of *Anaplasma* in southeastern and central Shanxi, China, Ixodid ticks were sampled from sheep and cattle host animals in 11 different geographic regions during 2022–2024. These tick samples were then subjected to *Anaplasma* detection via nested PCR combined with partial *16S rRNA* gene sequencing. The analysis revealed that 246 of 350 ticks were positive for *Anaplasma*. Among the survey areas, the prevalence rates of *Anaplasma* infection were 67.50% (27/40) in Wangjiazhuang Village, 74.19% (46/62) in Baitupo Village, 52.38% (11/21) in Daxigou Village, 66.67% (26/39) in Matian Town, 80.95% (34/42) in Zhuanghe Village, 75.00% (30/40) in Siyuan Village, 68.75% (11/21) in Xiwangyong Village, 70.83% (17/24) in Dongsitou Village, 76.19% (16/21) in Taling, 73.68% (14/19) in Houbu Village, 66.67% (14/21) in Shipan Village. The prevalence of *Anaplasma* is significantly higher in female ticks compared to males (89.73% *vs* 11.49%, *χ*^2^ = 191.614, *p* < 0.001). Sequencing results revealed that this study identified four species of *Anaplasma* namely *Anaplasma phagocytophilum*, *Anaplasma ovis*, *Anaplasma marginale* and *Anaplasma bovis*. Meanwhile, four strains belonging to the order Rickettsiales were also detected and named “Uncultured Rickettsiaceae bacterium”. Phylogenetic tree analysis examined the clustering and genetic relationships between the identified four *Anaplasma* species and other *Anaplasma* species available in the NCBI database. The Rickettsiaceae bacteria obtained in this study were also included in the phylogenetic tree construction, and they were found to form the out group as the root of the tree. Haplotype phylogeographic dynamics provided an in-depth exploration of the subtle evolutionary differences among the same species of *Anaplasma* and revealed their evolutionary pathways. It was shown that the evolutionary paths of *A. phagocytophilum*, *A. ovis*, and *A. bovis* were more complex compared to that of *A. marginale*. The 110 *A. phagocytophilum* sequences obtained in this study were classified into 13 haplotypes, while the 36 *A. ovis* sequences were grouped into 10 haplotypes. *A. bovis* and *A. marginale* each exhibited a single haplotype, as only one sequence has been obtained for each of them. In conclusion, a high prevalence of *Anaplasma* infection was observed in ticks from southeastern and central Shanxi, China. This finding lays a theoretical foundation for the formulation and implementation of relevant prevention and control measures in the study area. Continuous monitoring and epidemiological surveys of pathogens in host animals will be required in the future.

## Introduction

*Anaplasma* is a tick-borne hemopathogen in the order Rickettsiales, family Anaplasmataceae (Monyama et al., 2025). Since discovered in 1910, it primarily includes six species: *A. phagocytophilum*, *A. marginale*, *A. ovis*, *A. bovis*, *A. centrale*, and *Anaplasma platys* (Kolo, 2023). These *Anaplasma* species mainly cause diseases in vertebrates; in particular, *A. phagocytophilum* and *A. bovis* can also cause human infections (Lin et al., 2023). Human Granulocytic Anaplasmosis (HGA) is a common zoonotic disease caused by *A. phagocytophilum*. It typically presents with mild, self-limiting symptoms but may progress to severe disease and even be fatal (MacQueen and Centellas, 2022). Influenced by global climate and environmental changes, the number of HGA infections has been increasing annually (Acosta-España et al., 2025). The clinical symptoms of animal infected with *Anaplasma* are also known as Tick-Borne Fever (TBF), as exhibiting manifestations such as high fever, loss of appetite, emaciation, lethargy and the inhibition of innate immunity (Ge et al., 2016; Shahzadi et al., 2025).

The distribution of *Anaplasma* is closely linked to the geographical ranges of tick vectors and host animals (Qin et al., 2018; Levin et al., 2021), and the range is centered primarily in Asia, with extensions reaching into Europe and Africa. (Karshima et al., 2023; Huang et al., 2025). In China, *Anaplasma* is primarily distributed in the northeastern and northwestern regions, commonly found in mountainous, forests, grasslands, hills, and forms regional transmission through the activity range of host animals (Wei et al., 2016; Lu et al., 2022; Han et al., 2024). A single *A. phagocytophilum*-positive tick was identified in Shanxi Province (Yan et al., 2021). By contrast, Yang et al. detected no *A. phagocytophilum* in 600 ticks collected from Qi County in central Shanxi Province (Yang et al., 2021). In host animal surveillance, Zhang et al. found *Anaplasma* in 25 out of 32 sheep blood samples from Shanxi, with an infection rate of 78.1% (Zhang et al., 2016). Studies on the detection of tick-borne *Anaplasma* species in Shanxi Province remain limited.

Shanxi Province is situated in North-Central China, and the whole province lies in the transitional zone between temperate monsoon and continental climate. Reports indicate a rising temperature trend across Shanxi Province, accompanied by increases in both warm days and warm nights (Fan et al., 2012). Yangquan, located in central Shanxi Province, and Changzhi, in southeastern Shanxi, both border the Taihang Mountains, featuring largely mountainous landscapes and abundant natural epidemic foci. Sheep, cattle, and other livestock are widely raised in this area, and the region also harbors a high diversity of wildlife hosts. These conditions provide an potential environmental factors for the reproduction and activity of ticks, which may consequently enhance the transmission efficiency and epidemic potential of the *Anaplasma*. Therefore, conducting related research is of great significance for clarifying the transmission mechanisms of *Anaplasma*, tracing the spread of zoonotic diseases, and safeguarding public health.

Nowadays, the detection sensitivity and identification efficiency of *Anaplasma* have been remarkably enhanced, which is largely attributed to the widespread adoption of modern molecular biology techniques, including polymerase chain reaction (PCR), real-time quantitative PCR (qPCR), high-throughput sequencing, and loop-mediated isothermal amplification (LAMP), CRISPR/Cas systems (Yalcindag et al., 2024; Alsultan et al., 2025). Modern molecular biology techniques facilitate the screening of hosts infected with *Anaplasma* using only small volumes of samples. The *16S rRNA* gene is commonly used for the detection of *Anaplasma*, along with other specific genes such as *gltA*, *groEL*, *msp2*, *msp4* and *23S rRNA* (Dreghiciu et al., 2025). Nevertheless, the intricate genetic background of *Anaplasma*, its broad host range, and its ecological interactions with vector ticks still present substantial challenges for the effective early-stage screening, surveillance and prevention of the disease.

In our study, molecular detection and a comprehensive systematic analysis of the genetic evolution characteristics of *Anaplasma* in ixodid ticks will be conducted, aiming to provide basic data support for disease prevention and control efforts in Shanxi Province.

## Materials and methods

### Tick collection

Ticks were collected using tweezers from the ears, abdomen, and other body parts of animals, then placed in separate microcentrifuge tubes for preservation, subsequent morphological identification, and pathogen detection. Sampling was taken during the warmer months (April–September) to ensure the acquisition of representative ticks. Between 2022 and 2024, tick sampling sites were randomly selected across four cities in the southeastern and central regions of Shanxi Province, China: Changzhi, Yangquan, Jinzhong, and Yuncheng. The collection sites and sample sizes were as follows: Wangjiazhuang Village (Yangquan City suburbs, *n* = 40), Baitupo Village (Yu County, Yangquan City, *n* = 62), Daxigou Village (Yuanqu County, Yuncheng City, *n* = 21), Matian Town (Zuoquan County, Jinzhong City, *n* = 39), Zhuanghe Village (Pingshun County, Changzhi City, *n* = 42), Siyuan Village (Qinyuan County, Changzhi City, *n* = 40), Xiwangyong Village (Qinyuan County, Changzhi City, *n* = 21), Dongsitou Village (Pingshun County, Changzhi City, *n* = 24), Taling (Luzhou District, Changzhi City, *n* = 21), Houbu Village (Xiangyuan County, Changzhi City, *n* = 19), and Shipan Village (Wuxiang County, Changzhi City, *n* = 21). These 11 locations are situated between latitude 35°20′–38°05′N and longitude 110°31′–113°38′E. Ticks collected from Daxigou Village and Matian Town were parasitic on cattle, while those from the other nine locations were sheep-infesting ticks. Tick collection methods remained consistent across regions and years. Both adult and sub-adult sheep and cattle aged 1–4 years were selected, with 5–10 ticks collected from each animal.

The morphological characteristics of ticks were observed under a microscope, with focus on the basis capitalism, palp rings, scutum, spiracular plates and body profile, so as to preliminary classification using taxonomic keys (Namgyal et al., 2021). All the collected ticks were confirmed to be adult, with the female individuals exhibiting a state of full engorgement. Representative samples were selected for mitochondrial cytochrome c oxidase subunit I (COI) gene sequencing to complete molecular identification. Tick samples were then stored at −80 °C until further analysis.

### *Anaplasma* detection and sequencing

Ticks were rinsed 3–5 times with distilled water (5 min soaking per rinse), then air-dried gently under sterile conditions at room temperature for subsequent analysis. Each tick was placed individually into a 2 mL grinding tube, to which 200 μL precooled PBS buffer and three 2-mm-diameter steel balls were added. The mixture was ground thoroughly at 1800 rpm for 6 cycles; each cycle consisted of 60 s of grinding and a subsequent 10-s pause. Genomic DNA was extracted from each tick using the TIANamp Micro DNA Kit (DP316, Tiangen Biotech, Beijing) in accordance with the manufacturer’s instructions.

Nested PCR was conducted to amplify the partial *16S rRNA* gene of *Anaplasma.* The primers for the two consecutive amplification cycles were described in previous study (Zhang et al., 2013). The primary cycle used forward primer 5′-TTG AGA GTT TGA TCC TGG CTC AGA ACG–3′ and reverse primer 5′-CAC CTC TAC ACT AGG AAT TCC GCT ATC -3′; the secondary cycle used forward primer 5′-GTC GAA CGG ATT ATT CTT TAT AGC TTG-3′ and reverse primer 5′-TAT AGG TAC CGT CAT TAT CTT CCC TAC-3′. To ensure high amplification fidelity, the reaction mixture was carefully optimized using TaKaRa ExTaq polymerase Mix (RR001, TaKaRa Bio, Beijing). PCR amplification was performed in a total reaction volume of 20 μL. Each reaction consisted of 10 μL of 2 × Taq Mix, 0.8 μL of each forward and reverse primer (10 μM), 1 μL of template DNA (50–200 ng), and nuclease-free water to make up the final volume. The same reaction system was applied for both rounds of nested PCR amplification. Negative and positive controls were included in all PCR experiments. The PCR amplification conditions were established as follows: an initial denaturation step at 94 °C for 10 min; followed by 35 cycles of amplification, each consisting of denaturation at 94 °C for 30 s, annealing at 56 °C for 30 s, and extension at 72 °C (90 s for the primary amplification cycle and 45 s for the secondary amplification cycle); and a final extension step at 72 °C for 10 min. Amplicons were identified by 1.5% agarose gel electrophoresis, and then the highly specific positive samples were sent to Sangon Biotech (Shanghai) for post-amplification sequencing.

### Phylogenetic analysis

The obtained sequencing data were compared with available reference *16S rRNA* gene sequences of *Anaplasma* from GenBank using the online BLAST tool^1^ and subsequently submitted to the NCBI database. For phylogenetic tree construction, the nucleotide sequences obtained in this study, together with homologous *16S rRNA* gene sequences retrieved from the NCBI GenBank public database, were included in the analysis. All nucleotide sequences were first aligned using ClustalW to maximize positional homology. Phylogenetic reconstruction was then performed within the Maximum likelihood (ML) framework to infer evolutionary history, with node confidence assessed by 1,000 bootstrap repetitions in MEGA-X software.

### Haplotype analysis

DnaSP 6.12.03 software was used to analyze nucleotide and haplotype diversity by identifying conserved sequences and polymorphic sites. The following parameters were assessed: the number of sequences (*n*), number of polymorphic sites (*s*), number of haplotypes (*h*), nucleotide diversity (*π*), average number of nucleotide differences (*κ*), haplotype diversity (Hd), and variance. Arlequin 3.5.2.2 software was employed for the automatic clustering of homologous sequences, with the resultant data matrix being used for haplotype network construction. The phylogeographic pattern of *Anaplasma* was mapped in Population Analysis with Reticulate Trees (PopART) version 1.7 by combining matrix analysis and application of the TCS (Templeton-Crandall-Sing) algorithm.

### Statistical analysis

The Chi-square test or Fisher’s exact test was employed to compare the differences in *Anaplasma* infection positive rates among different groups, with subsequent Bonferroni correction for multiple comparisons. All statistical analyses were conducted with SPSS Version 22.0 (SPSS Inc., Chicago, IL, United States), and the significance threshold was set at *p* < 0.05 for all tests.

## Results

### Tick collection

During the period of 2022–2024, a total of 350 tick samples were collected via host-attachment sampling at the 11 survey sites from the southeastern and central regions of Shanxi Province, China. Among them, there are 290 sheep-infesting ticks and 60 cattle-infesting ticks. The number of female ticks is 263, compared with 87 male ticks. Preliminary morphological identification of tick species and life stages were conducted using stereomicroscopy with reference to existing taxonomic keys. Molecular identification verified that all collected ticks were *Haemaphysalis longicornis*, one of the most prevalent ixodid tick species in northern China.

#### *Anaplasma* infection

To investigate the infection of *Anaplasma* in ticks from southeastern and central Shanxi, a systematic survey was conducted across 11 locations. Species-specific PCR targeting partial *16S rRNA* gene (370 bp) was employed for *Anaplasma* detection. As shown in Table 1, the infection rates were 67.50% (27/40) in Wangjiazhuang Village, 74.19% (46/62) in Baitupo Village, 52.38% (11/21) in Daxigou Village, 66.67% (26/39) in Matian town, 80.95% (34/42) in Zhuanghe Village, 75.00% (30/40) in Siyuan Village, 68.75% (11/21) in Xiwangyong Village, 70.83% (17/24) in Dongsitou Village, 76.19% (16/21) in Taling, and 73.68% (14/19) in Houbu Village, 66.67% (14/21) in Shipan Village (Table 1). *Anaplasma* infection rates varied in distribution but did not differ significantly among regions statistically (*χ^2^* = 10.598, *p* = 0.390). A representative nucleic acid gel image was provided in Supplementary Figure S1. Notably, the highest infection rate (80.95%) was observed in Zhuanghe Village, while Daxigou Village had the minimum infection rate (52.38%). Across all sampling locations, *Anaplasma* infections were predominantly identified in female ticks, with an overall infection rate of 89.73% (236/263). In particular, the *Anaplasma* infection rate among female ticks collected from Zhuanghe Village, Taling Village, Houbu Village and Shipan Village reached 100%. By sharp contrast, male ticks exhibited a markedly lower infection rate, accounting for only 11.49% (10/87, Supplementary Table S1). The *Anaplasma* infection rates differed significantly between female and male ticks (*χ^2^* = 191.614, *p* < 0.001). Moreover, the infection rate in sheep-infesting ticks (72.07%) was slightly higher than that in cattle-infesting ticks (67.50%), but no statistically significant difference was observed (*χ^2^* = 2.576, *p* = 0.109) (Supplementary Table S2).

**Table 1:** Polymerase chain reaction-based detection of *Anaplasma* infection prevalence across 11 different sampling sites.

Sampling sites	Tick hosts	No. captured and tick types	No. PCR positive	Positivity rate (%)
Wangjiazhuang village, Suburban district, Yangquan city	Sheep	Female (26)	Female (24)	Female (92.31)
		Male (14)	Male (3)	Male (21.43)
		Total (40)	Total (27)	Total (67.50)
Baitupo village, Yu county, Yangquan city	Sheep	Female (48)	Female (44)	Female (91.67)
		Male (14)	Male (2)	Male (14.29)
		Total (62)	Total (46)	Total (74.19)
Daxigou village, Yuanqu county, Yuncheng city	Cattle	Female (14)	Female (11)	Female (78.57)
		Male (7)	Male (0)	Male (0.00)
		Total (21)	Total (11)	Total (52.38)
Matian town, Zuoquan county, Jinzhong city	Cattle	Female (32)	Female (26)	Female (81.25)
		Male (7)	Male (0)	Male (20.00)
		Total (39)	Total (26)	Total (66.67)
Zhuanghe village, Pingshun county, Changzhi city	Sheep	Female (32)	Female (32)	Female (100.00)
		Male (10)	Male (2)	Male (0.00)
		Total (42)	Total (34)	Total (80.95)
Siyuan village, Qinyuan county, Changzhi city	Sheep	Female (32)	Female (28)	Female (87.50)
		Male (8)	Male (2)	Male (25.00)
		Total (40)	Total (30)	Total (75.00)
Xiwangyong village, Qinyuan county, Changzhi city	Sheep	Female (16)	Female (11)	Female (68.75)
		Male (5)	Male (0)	Male (0.00)
		Total (21)	Total (11)	Total (68.75)
Dongsitou village, Pingshun county, Changzhi city	Sheep	Female (19)	Female (16)	Female (84.21)
		Male (5)	Male (1)	Male (20.00)
		Total (24)	Total (17)	Total (70.83)
Taling village, Luzhou district, Changzhi city	Sheep	Female (16)	Female (16)	Female (100.00)
		Male (5)	Male (0)	Male (0.00)
		Total (21)	Total (16)	Total (76.19)
Houbu village, Xiangyuan county, Changzhi city	Sheep	Female (14)	Female (14)	Female (100.00)
		Male (5)	Male (0)	Male (0.00)
		Total (19)	Total (14)	Total (73.68)
Shipan village, Wuxiang county, Changzhi city	Sheep	Female (14)	Female (14)	Female (100.00)
		Male (7)	Male (0)	Male (0.00)
		Total (21)	Total (14)	Total (66.67)

##### Identification and phylogenetic analysis of *Anaplasma* species

Blast analysis of *16S rRNA* gene sequences on NCBI revealed that 110 sequences shared 99.16%–100% homology with the reference strain of *A. phagocytophilum* in the NCBI GenBank. Thirty-six sequences exhibited 99.16%–100% homology with *A. ovis*. One sequence obtained in Siyuan Village showed 99.16% homology with *A. marginale* (Accession No. OM065783.1), while another one sequence obtained in Houbu Village demonstrated 99.16% homology with *A. bovis* (JN558822.1). Additionally, three strains from cattle-infesting ticks in Daxigou Village and one from sheep-infesting ticks in Baitupo Village showed high homology to Rickettsia strains (JQ701668.1 and OQ326846.1), and were subsequently named “Uncultured Rickettsiaceae bacterium”. After sequence assembly and quality control, 152 *Anaplasma 16S rRNA* sequences have been deposited in GenBank (PX622722-PX622873). As detailed in Supplementary Table S3, the serial numbers, *Anaplasma* species names, and corresponding isolation sites were provided. The remaining 94 samples were not identifiable due to inferior Sanger-sequence quality or co-infections with other species. *Anaplasma* species identification by partial *16S rRNA* sequencing was successful in 61.79% (152/246) of infected ticks.

**Figure 1:**
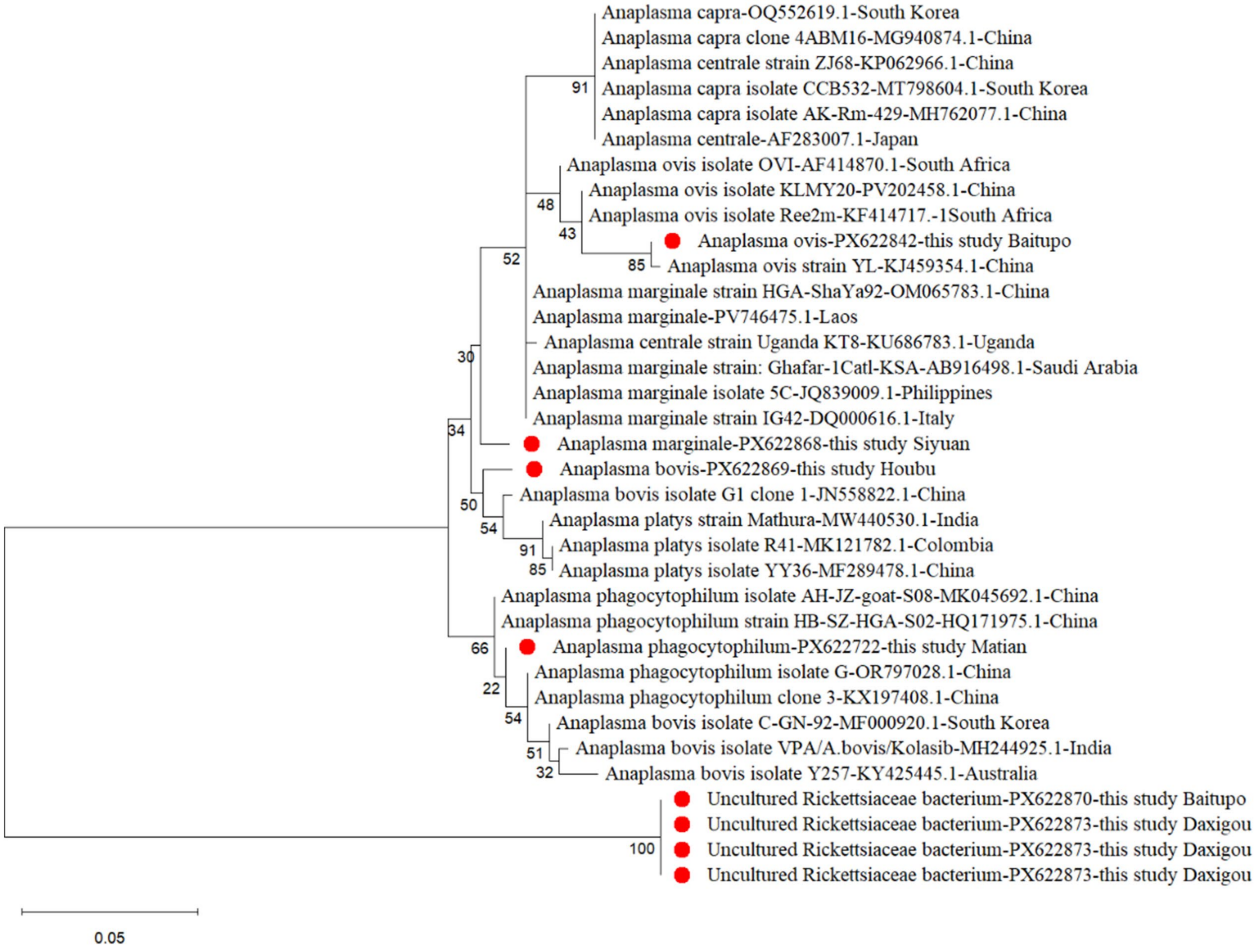
A maximum likelihood (ML) phylogenetic analysis of *Anaplasma* spp. based on partial *16S rRNA* gene sequences. The bootstrap repetitions are 1,000. Representative sequences in this study are indicated by red dots. Each entry is annotated with the corresponding *Anaplasma* species, NCBI accession number, and geographic origin.

**Figure 2:**
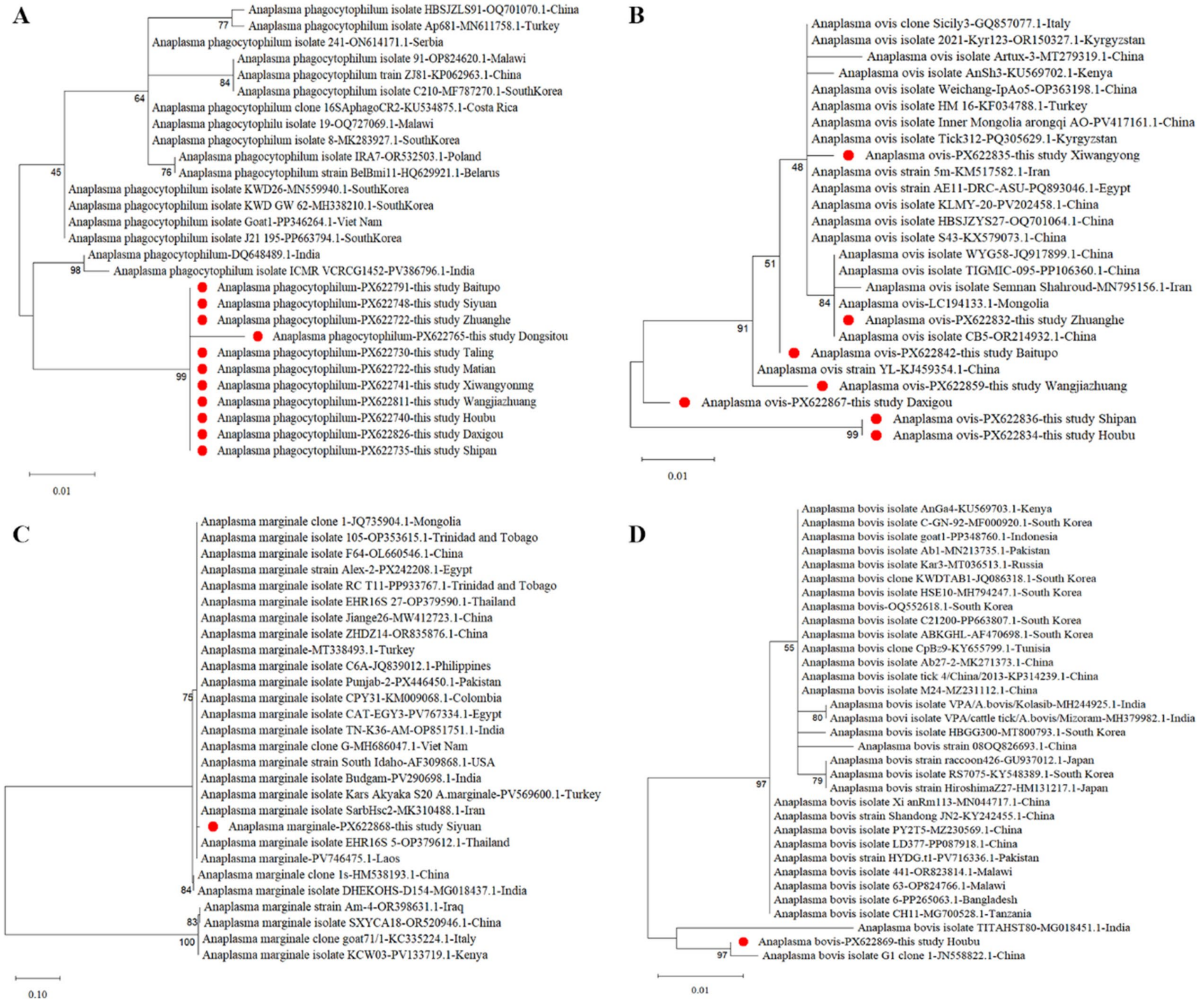
Four Maximum likelihood (ML) Phylogenetic trees of partial *16S rRNA* gene sequences of *Anaplasma phagocytophilum* **(A)**, *Anaplasma ovis* **(B)**, *Anaplasma marginale* **(C)**, and *Anaplasma bovis* **(D)**. The bootstrap repetitions are 1,000. Representative sequences in this study are indicated by red dots. Each entry is annotated with the corresponding *Anaplasma* species, NCBI accession number, and geographic origin.

To clarify the phylogenetic relationships of *Anaplasma* identified in this study with other species within the genus *Anaplasma*, a phylogenetic tree was constructed using the Maximum-Likelihood method in MEGA-X software. The analysis of the first phylogenetic tree included one *16S rRNA*-positive sample from each of the four obtained *Anaplasma* species and the four uncultured Rickettsiaceae bacteria, supplemented with 27 representative *Anaplasma 16S rRNA* gene sequences retrieved from GenBank (Figure 1). Phylogenetic analysis revealed that the *A. phagocytophilum* obtained in this study was closely related to isolates from Hubei (HQ171975.1), Anhui (MK045692.1, OR797028.1), and Jilin (KX197408.1) in China, indicating high homology with multiple domestic strains. Furthermore, it was shown that the *A. phagocytophilum* strains isolated from 11 different survey regions in this study exhibited close genetic relatedness (Figure 2A). The *A. ovis* identified in this study clustered with *A. ovis* from *Dermacentor marginatus* in Xinjiang, China (KJ459354.1) in Figure 1. As shown in Figure 2B, the *A. ovis* strains obtained in seven survey areas exhibit a certain degree of evolutionary diversity. Among them, the strains isolated from Shipan and Houbu show a more distant evolutionary relationship compared to those from other five regions. The first phylogenetic tree revealed that *A. marginale* identified in this study was closely related to one strain isolated from Xinjiang, China (OM065783.1), which was consistent with the results shown in Figure 2C. The *A. bovis* strain isolated from Houbu Villages clustered together with an *A. bovis* strain from Guizhou, China (JN558822.1) (Figure 1), and also exhibit a close evolutionary relationship with one strain isolated from India (MG018451.1) (Figure 2D). Notably, the four Rickettsia strains constituted an out group at the root of the phylogenetic tree, which represents a primitive, divergent lineage distinct from other clades in the tree (Figure 1).

##### Geophylogenetic network of *Anaplasma* strains

**Figure 3:**
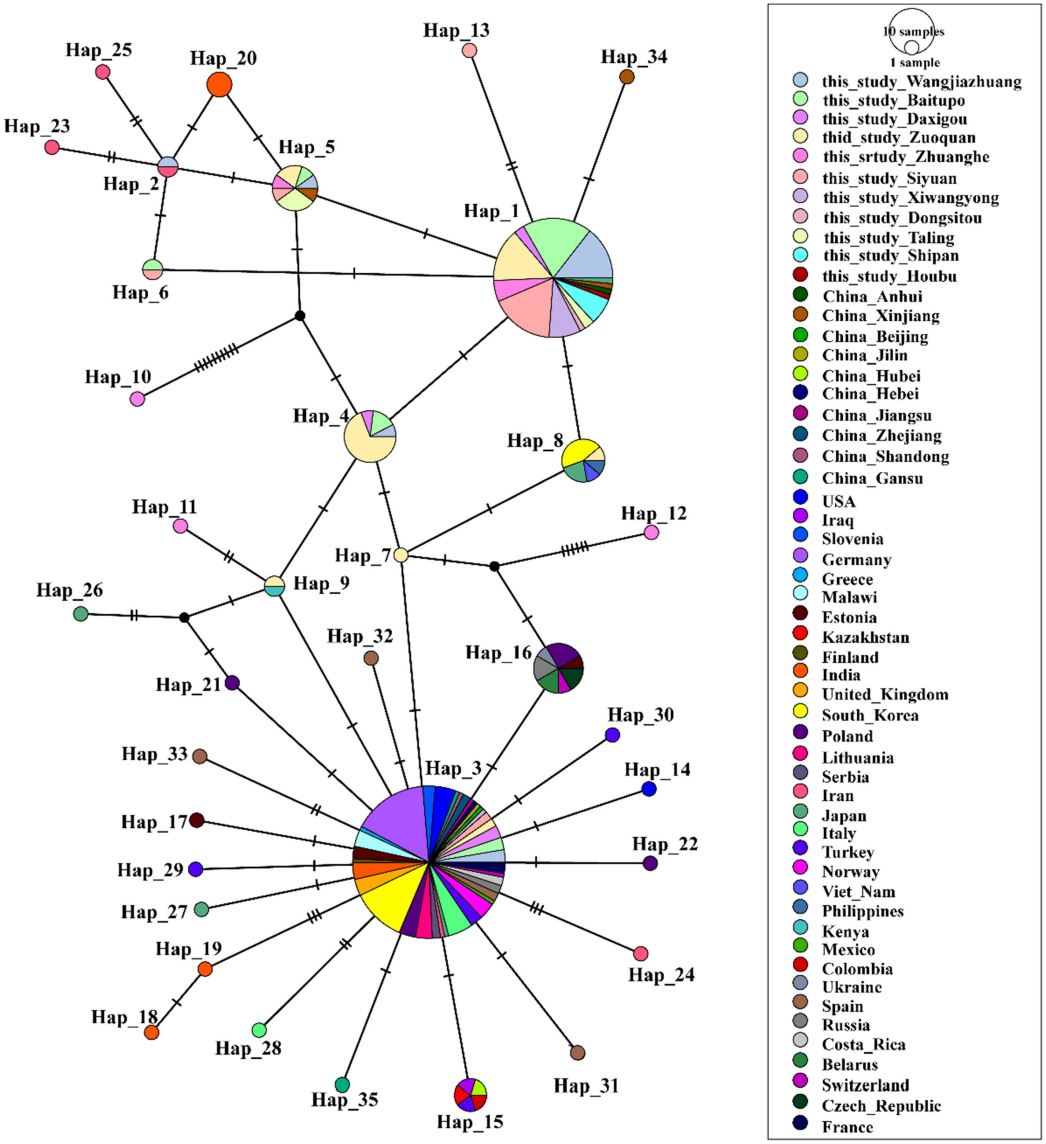
Phylogeographic network of *A. phagocytophilum 16S rRNA* haplotypes (Templeton-Crandall-Sing algorithm). Each color corresponds to a distinct geographic location. Each colored circle represents a haplotype, and the size of the circle corresponds to the number of that haplotype. Black dots stand for the inferred haplotypes that may exist but for which no samples were obtained. Short vertical lines on the long connecting lines indicate the number of base substitutions required for one haplotype to convert to its linked haplotype. *A. phagocytophilum* detected in Wangjiazhuang was classified into Hap1 (*n* = 9), Hap2 (1), and Hap3 (3), Hap4 (1), Hap5 (1), that from Baitupo to Hap1 (13), Hap3 (3), Hap4 (2), Hap5 (1), and Hap6 (1), that from Daxigou to Hap1 (2), Hap3 (3), and Hap4 (1), that from Zuoquan to Hap1 (10), Hap3 (2), Hap4 (9), Hap5 (1), Hap7 (1), Hap8 (1), and Hap9 (1), that from Zhanghe to Hap1 (4), Hap5 (1), Hap10 (1), Hap11 (1), and Hap12 (1), that from Siyuan to Hap1 (12), Hap3 (2), Hap5 (1), Hap6 (1), and Hap13 (1), that from Xiwangyong to Hap1 (6) and Hap3 (1), that from Dongsitou to Hap1 (1), that from Taling to Hap1 (2) and Hap5 (3), that from Shipan to Hap1 (5), that from Houbu to Hap1 (1).

To perform a haplotype evolution analysis, we selected representative *A. phagocytophilum* sequences from the NCBI database and conducted a combined analysis incorporating all 110 *A. phagocytophilum* sequences generated herein. A total of 35 haplotypes were identified (*n* = 263, *S* = 46, *h* = 35, *π* = 0.01256, *κ* = 2.110, Hd = 0.742 ± 0.00045) (Figure 3). The corresponding hosts and collection sites for each haplotype were detailed in Supplementary Table S4. Based on TCS haplotype network analysis, haplotype 3 (Hap 3) was identified as the predominant haplotype of *A. phagocytophilum*, exhibiting a global distribution pattern and being widely detected across Asia, Europe, North America, and Africa. Besides, Hap3 has a remarkably broad host range, covering diverse ixodid tick species (*Ixodes ricinus*, *Ixodes persulcatus*, and *H. longicornis*) as well as multiple mammalian hosts, including humans, cattle, horses, and deer. Hap1 was derived from Hap3 via Hap4 to Hap7 through multiple mutations, and a large number of sequences obtained in this study fall into this haplotype, exhibiting significant regional clustering.

**Figure 4:**
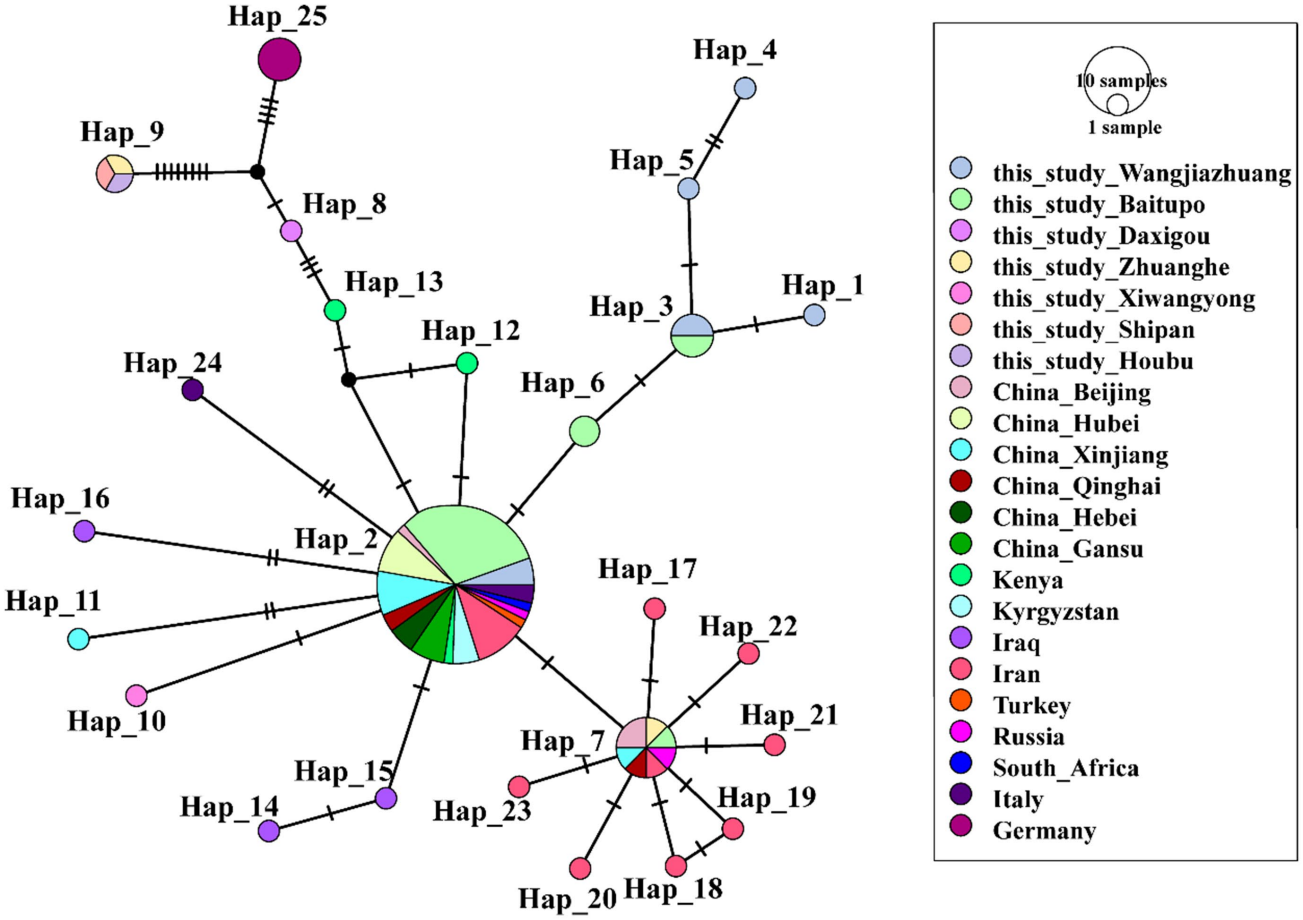
Phylogeographic network of *A. ovis 16S rRNA* haplotypes (Templeton-Crandall-Sing algorithm). Each color corresponds to a distinct geographic location. *A. ovis* detected in Wangjiazhuang was classified into Hap1 (*n* = 1), Hap2 (3), Hap3 (2), Hap4 (1) and Hap5 (1), that from Baitupo to Hap2 (17), Hap3 (2), Hap6 (2) and Hap7 (1), that from Daxigou to Hap8 (1), that from Zhanghe to Hap7 (1) and Hap9 (1), that from Xiwangyong to Hap10 (1), that from Shipan to Hap9 (1), that from Houbu to Hap9 (1).

The sequences of *A. ovis* obtained in this study were combined with reference sequences from NCBI for comprehensive analysis, leading to the identification of 25 haplotypes (*n* = 95, *S* = 32, *h* = 25, *π* = 0.01028, *κ* = 2.633, Hd = 0.658 ± 0.00308) (Figure 4, Supplementary Table S5). Haplotype network analysis revealed that Hap2 was the core haplotype of *A. ovis*, with most sequences from this study clustering within it.

**Figure 5:**
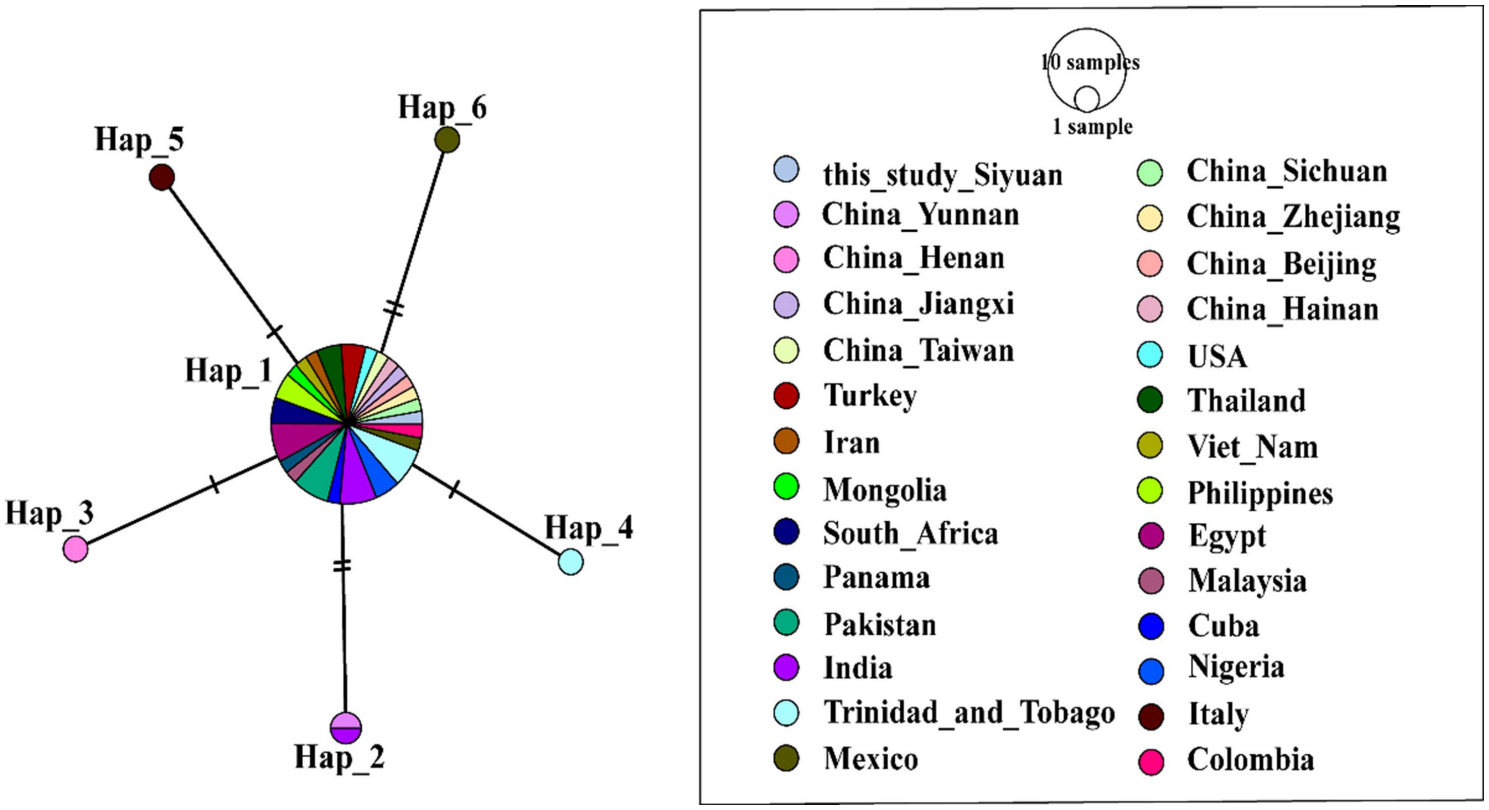
Phylogeographic network of *A. marginale 16S rRNA* haplotypes (Templeton-Crandall-Sing algorithm). Each color corresponds to a distinct geographic location. *A. marginale* detected in Siyuan was classified into Hap1 (*n* = 1).

The indentifed *A. marginale* in this study was analyzed together with reference sequences collected from NCBI, resulting in the identification of 6 haplotypes (*n* = 44, *S* = 7, *h* = 6, *π* = 0.00365, *κ* = 0.405, Hd = 0.256 ± 0.00746) (Figure 5, Supplementary Table S6). Haplotype network analysis revealed that *A. marginale* Hap1 acts as the core haplotype, from which five haplotypes (Hap2, 3, 4, 5, 6) are directly derived via minor genetic variations. The only *A. marginale* sequence obtained in this study belongs to Hap2. These low-frequency haplotypes, derived from the core haplotype, have each formed independent evolutionary branches. Their genetic structure has tended to stabilize, with no further mutations or evolutionary changes occurring.

**Figure 6:**
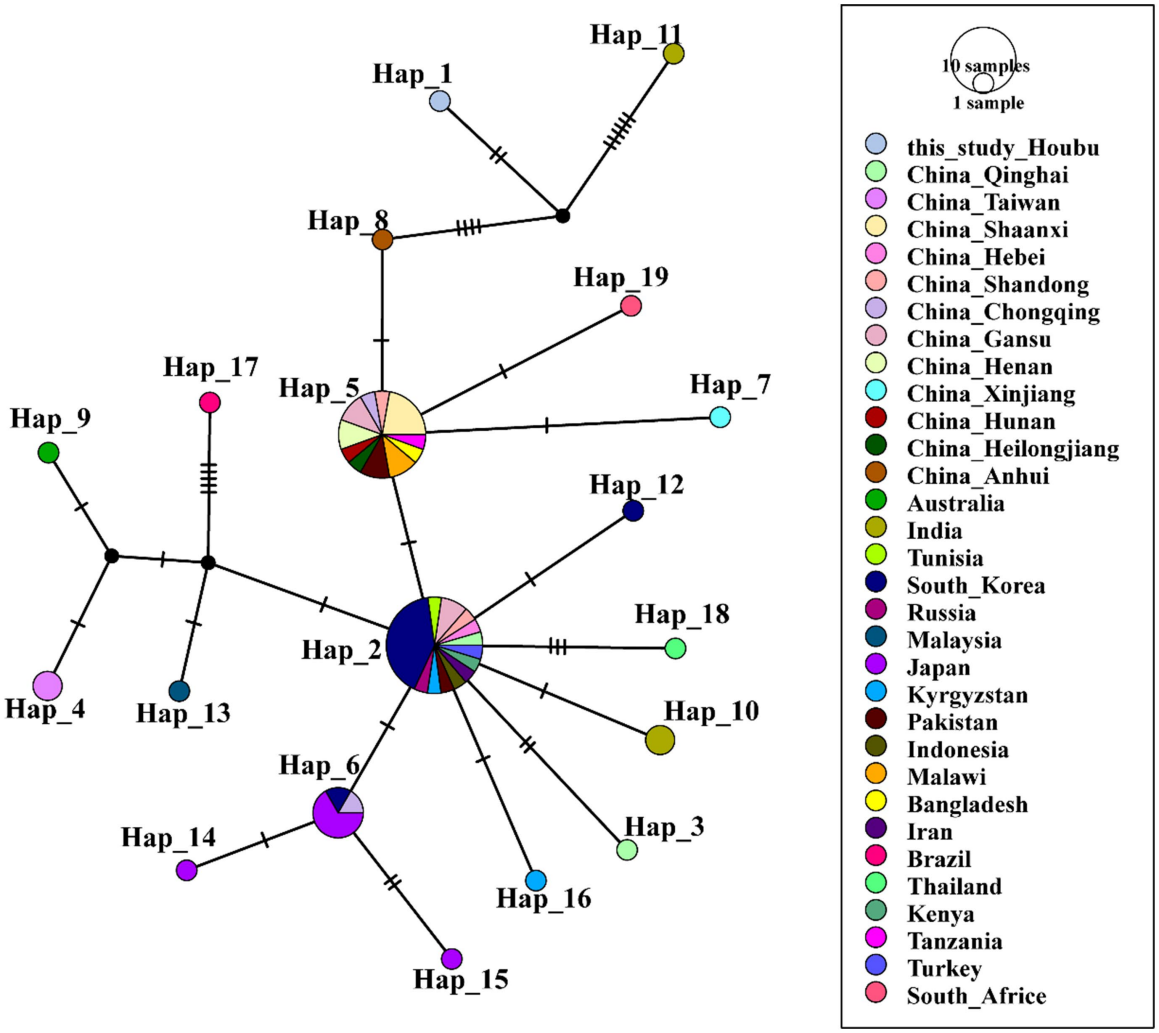
Phylogeographic network of *A. bovis 16S rRNA* haplotypes (Templeton-Crandall-Sing algorithm). Each color corresponds to a distinct geographic location. *A. bovis* detected in Houbu was classified into Hap1 (*n* = 1).

After integrating and analyzing the only *A. bovis* sequence in this study with NCBI reference sequences, a total of 19 haplotypes were identified (*n* = 61, *S* = 34, *h* = 19, *π* = 0.00739, *κ* = 2.225, Hd = 0.801 ± 0.00126) (Figure 6, Supplementary Table S7). Haplotype network analysis uncovered that Hap 2 and Hap 5 acted as the two core haplotypes, connected to each other by only one mutational step. The *A. bovis* sequence obtained in Houbu Village belonged to Hap 1, which evolved from the core Hap 5 via the derived mutation of Hap8.

## Discussion

With the intensification of global climate change and cross-border activities, the geographical distribution range of ticks continues to expand, and the resulting vector-borne infection issues have become a significant public health challenge (Byun et al., 2025). A comprehensive understanding of the biological characteristics, transmission dynamics, and pathogenic mechanisms of tick-borne pathogens is crucial for enhancing tick surveillance and management, as well as for effectively interrupting transmission pathways and preventing zoonotic diseases.

As an important obligate intracellular bacterium, *Anaplasma* relies on ixodid ticks as its primary reservoir hosts and transmission vectors. *Anaplasma* can infect vertebrates such as humans, livestock, and wildlife, leading to the occurrence of *anaplasma*-related diseases (Cheng et al., 2025). Although *Anaplasma* infection is common in both vertebrates and ticks across China, knowledge regarding the distribution of this pathogen in ticks from Shanxi, remains limited. This study aims to investigate the distribution of *Anaplasma* in Shanxi, China, where ticks collected in the present study have been found to have a high positivity rate for *Anaplasma* spp. Based on nested PCR and *16S rRNA*-specific gene sequencing, four *Anaplasma* species were identified in the surveyed areas, namely *A. phagocytophilum*, *A. ovis*, *A. marginale*, and *A. bovis*. This finding signifies that *H. longicornis* in these regions act as potential vectors for *Anaplasma* and implies a considerable risk of anaplasmosis to local livestock and wildlife populations. *H. longicornis* is an important vector in China and the dominant tick species in Shanxi Province (Zhao et al., 2021). It harbors the major species of the family Anaplasmataceae, including *A. phagocytophilum*, *Anaplasma capra*, *A. platys*, *A. ovis*, *Ehrlichia canis*, *Ehrlichia chaffeensis*, and *Ehrlichia ewingii* (Zhao et al., 2020). Among these, *A. phagocytophilum* was the most prevalent. Our observation confirmed this current prevalence and extends the known distribution range of *Anaplasma* in China. These findings are generally consistent with the *Anaplasma* species identified in *H. longicornis* from neighboring Shaanxi, Henan, and Hebei provinces (Cao et al., 2021; Yan et al., 2021; Teng et al., 2023). Particularly, this study supports the findings of a previous investigation into *Anaplasma* infections in animal hosts in Taiyuan and Jincheng cities of Shanxi Province. That study documented a high prevalence of *Anaplasma* in sheep, as well as four cases of coinfection with *A. phagocytophilum*, *A. ovis*, and *A. bovis* (Zhang et al., 2016). Furthermore, the prevalence of *Anaplasma* infection in our surveyed area was significantly higher in female ticks than in males. This disparity is hypothesized to be associated with biological differences between male and female ixodid ticks, as female ticks display greater blood-feeding volumes and larger genomic content than males (Yuan et al., 2025), thereby supplying a greater initial dose of intracellular pathogen and a nutrient-abundant environment for its replication. Besides, the parthenogenetic reproduction mechanism of *H. longicornis* also appears to subtly account for such heterogeneous aggregation. Interestingly, in many studies on tick-borne *Borrelia* sp., female ticks have also been found to harbor a higher pathogen load than male ticks (Alekseev et al., 1999; Koloski et al., 2025). Additionally, the infection rate of *Anaplasma* in sheep-infesting ticks was slightly higher than that in cattle-infesting ticks, which may also be related to the fact that local sheep have longer grazing periods than cattle, making them more susceptible to tick infestation. Although the 11 surveyed regions share similar bioclimatic characteristics, the infection rate of *Anaplasma* still shows certain variations, potentially linked to sampling bias, the lifestyle of host animals, divergences in local grazing or agricultural practices. Ticks were found to harbor a diverse range of *Anaplasma* species, yet none of the surveyed animals showed any obvious clinical signs. It is therefore speculated that these *Anaplasma* strains have limited pathogenicity in ruminants and other domestic animals. Notably, data for this study were collected via a cross-sectional survey, which may only reflect pathogen detection status within the sampling window and cannot fully reveal the spatiotemporal heterogeneity of pathogen transmission dynamics. Future studies should expand the scale of sample collection and carry out multi-seasonal sampling surveys to clarify the prevalence patterns of *Anaplasma* in different hosts and regions, thereby revealing the true impact of anaplasmosis.

Phylogenetic analysis revealed that the *A. phagocytophilum* strains identified in this study formed a tight cluster with reference sequences of *A. phagocytophilum* deposited in GenBank, displaying branches with high sequence homology. This indicates that the *A. phagocytophilum* carried by ixodid ticks in Shanxi Province has a well-defined taxonomic status and is closely related to isolates from other regions in China. Notably, the *A. bovis* sequence obtained in this study, together with one *A. bovis* strain (JN558822.1) and three *A. platys* strains, formed a distinct cluster (Figure 1). This clustering pattern suggests a certain degree of evolutionary homology between *A. bovis* and *A. platys*, a phenomenon presumably driven by the complexity of evolutionary divergence in these two species or biases in sequence selection. The identified *A. marginale* formed an out group when compared with other reported *A. marginale* isolates in the comprehensive intragenic phylogenetic tree (Figure 1), and exhibited a greater interspecies evolutionary distance than that of *A. phagocytophilum*, *A. ovis*, and *A. bovis*. (Figure 2). This observation may be ascribed to two potential factors: first, the relatively short length of the sequence generated in this study imposed constraints on its clustering analysis with other *A. marginale* strains; second, the *A. marginale* isolated herein exhibited a high degree of genetic variation, which impeded its effective clustering with the reference sequences of *A. marginale* from the database.

Haplotype analysis further clarifies the evolutionary history of these four *Anaplasma* species and provides valuable insights into their population expansion processes as well as interspecies genetic exchange events (Jaarsma et al., 2019; Rar et al., 2021). The results showed that the detected *A. phagocytophilum* and *A. ovis* in this study had multiple haplotypes, and different haplotypes displayed a certain degree of geographical clustering in the phylogenetic analysis. This suggests that *Anaplasma* in this region may have diversified evolutionary pathways, which could be related to factors such as host migration, dispersal of tick vectors, and differences in ecological environments (Torina et al., 2010). In the haplotype analysis of *A. phagocytophilum*, we found that the sequences obtained in this study were mainly distributed in Hap1 and Hap3. Nearly all sequences of Hap1 were produced in this study, indicating that Hap1 may be the dominant epidemic lineage of *Anaplasma* in the Shanxi region of China. In the haplotype analysis of *A. ovis*, it was observed that the obtained *A. ovis* strains were predominantly distributed across two clades derived from the core haplotype hap2 (Figure 4, Supplementary Table S5). The first clade comprises Hap1, Hap3, Hap4, Hap5, and Hap6. These haplotypes correspond to strains predominantly isolated from Wangjiazhuang and Baitupo villages, collectively forming an independent clade. This finding indicates that the *A. ovis* strains from these two geographically adjacent villages within the same city share high genetic homology, reflecting a close evolutionary relationship between them. The second clade consists exclusively of Hap7, and some of the strains isolated from Baitupo Village and Zhuanghe Village belong to this haplotype. As a key lineage divergent from the core haplotype, it serves as an important secondary evolutionary hub for *A. ovis* haplotypes. Haplotype analysis of *A. marginale* revealed that this species harbored fewer haplotypes than the other three *Anaplasma* species, a feature suggestive of low genetic diversity and variation. This characteristic may also represent a population-specific trait of the *A. marginale* strains (Ierardi, 2025; Mauri Pablo et al., 2025). Evolutionary analysis of *A. bovis* haplotypes revealed that a single sequence isolated from Houbu village was classified into Hap1, which was genetically distant from the core haplotypes (Hap2 and Hap5). We infer that this *A. bovis* strain from Houbu village exhibits relatively high genetic variability. Overall, the haplotype analysis in this study covers a broad range of *Anaplasma* species from China and other global regions, yet its accuracy remains comparatively restricted due to the utilization of partial *16S rRNA* gene sequences. Future research should employ higher-resolution molecular markers, including multilocus sequence typing (MLST) and whole-genome single nucleotide polymorphism (SNP) analysis, to better characterize the population genetic structure and evolutionary dynamics of *Anaplasma*.

Based on the analysis of tick samples from the southeastern and central regions of Shanxi Province, this study conducted a comprehensive investigation on *Anaplasma*, focusing on infection status, species identification, phylogenetic relationships, and geographic phylogenetic networks features. It confirmed the presence of *Anaplasma* in the southeastern and central region of Shanxi Province, China, providing new data for understanding the transmission ecology of this pathogen. While this study has indicated the potential for tick-borne transmission, the available data are still very limited, and this should be acknowledged. Limitations including a small sample size, reliance on a single tick species, imbalanced sampling of male/ female ticks and cattle/ sheep-infesting ticks, as well as the lack of systematic seasonal surveillance and epidemiological investigations of host animals for tick-borne *Anaplasma*, may hinder a full assessment of the actual pathogen diversity and epidemic risk in this region.

## Conclusion

Overall, this study characterizes the prevalence of *Anaplasma* spp. in ticks from 11 districts of Shanxi Province, China, and reveals variations in infection rates across different sampling districts. The relatively high prevalence of *Anaplasma* spp. underscore the importance of conducting epidemiological investigations on both animals and vectors in Shanxi Province. This initiative aims to enhance the understanding of transmission dynamics among *Anaplasma*, ticks, and animals, thereby effectively reducing the economic losses caused by anaplasmosis in small ruminant production.

## Data Availability

The sequencing data that support this study are openly available in the NCBI database under accession number PX622722 to PX622873. All data supporting the findings of this study are available in the article and its supplementary information.

## Footnotes

1
https://blast.ncbi.nlm.nih.gov
