# Supplementary material for: Molecular detection and genetic diversity of Anaplasma in ticks from southeastern and central Shanxi, China
Source: Front Microbiol. 2026 Mar 10;17:1778059. doi: 10.3389/fmicb.2026.1778059 (PMC13008897; doi:10.3389/fmicb.2026.1778059)
Supplement: Supplementary file 1 [file Data_Sheet_1.docx]

Supplement Figure S1 Agarose gel image of *16S rRNA* gene amplification of *Anaplasma* spp. in tick samples. Lane M: DL2000 DNA ladder; Lane 1-24: PCR product of partial tick samples


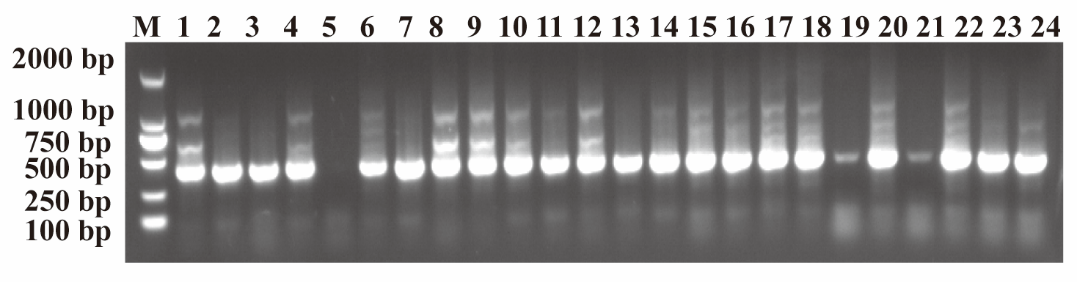


Supplement Table S1 The polymerase chain reactions (PCR) diagnose of *Anaplasma* spp. in female and male ticks

| Types | No.captured | No.PCR positive | positivity rate (%) |
| --- | --- | --- | --- |
| female ticks | 263 | 236 | 89.73 |
| male ticks | 87 | 10 | 11.49 |

Supplement Table S2 The polymerase chain reactions (PCR) diagnose of *Anaplasma* in different hosts

| Hosts | No.captured and Tick types | No.PCR positive | Positivity rate (%) |
| --- | --- | --- | --- |
| Cattle | female (46)  male (14)  total (60) | female (37)  male (0)  total (37) | female (80.43)  male (0.00)  total (67.50) |
| Sheep | female (217)  male (73)  total (290) | female (199)  male (10)  total (209) | female (91.70)  male (13.70)  total (72.07) |

Supplement Table S3 Sequence information of *Anaplasma* obtained in this study deposited in NCBI

| Species | Accession NO. | location-site |
| --- | --- | --- |
| *Anaplasma phagocytophilum* | PX622722- PX622729 | Zhuanghe, Shanxi, China |
| *Anaplasma phagocytophilum* | PX622730- PX622734 | Taling, Shanxi, China |
| *Anaplasma phagocytophilum*  *Anaplasma phagocytophilum* | PX622735- PX622739  PX622740 | Shipan, Shanxi, China  Houbu, Shanxi, China |
| *Anaplasma phagocytophilum* | PX622741- PX622747 | Xiwangyong, Shanxi, China |
| *Anaplasma phagocytophilum* | PX622748- PX622764 | Siyuan, Shanxi, China |
| *Anaplasma phagocytophilum* | PX622765 | Dongsitou, Shanxi, China |
| *Anaplasma phagocytophilum* | PX622766- PX622790 | Zuoquan, Shanxi, China |
| *Anaplasma phagocytophilum* | PX622791- PX622810 | Baitupo, Shanxi, China |
| *Anaplasma phagocytophilum* | PX622811- PX622825 | Wangjiazhuang, Shanxi, China |
| *Anaplasma phagocytophilum* | PX622826- PX622831 | Daxigou, Shanxi, China |
| *Anaplasma ovis* | PX622832, PX622833 | Zhuanghe, Shanxi, China |
| *Anaplasma ovis* | PX622834 | Houbu, Shanxi, China |
| *Anaplasma ovis* | PX622835 | Xiwangyong, Shanxi, China |
| *Anaplasma ovis* | PX622836 | Shipan, Shanxi, China |
| *Anaplasma ovis* | PX622837-PX622858 | Baitupo, Shanxi, China |
| *Anaplasma ovis* | PX622859-PX622866 | Wangjiazhuang, Shanxi, China |
| *Anaplasma ovis* | PX622867 | Daxigou, Shanxi, China |
| *Anaplasma marginale* | PX622868 | Siyuan, Shanxi, China |
| *Anaplasma bovis* | PX622869 | Houbu, Shanxi, China |
| uncultured *Rickettsiaceae* bacterium | PX622870 | Baitupo, Shanxi, China |
| uncultured *Rickettsiaceae* bacterium | PX622871-PX622873 | Daxigou, Shanxi, China |

Supplement Table S4 Haplotypes of *Anaplasma phagocytophilum* involved in figure 3

| Haplotype | Acc. no | Host | Site |
| --- | --- | --- | --- |
| Hap1 | PX622811 | *Haemaphysalis longicornis* | China-Shanxi-Wangjiazhuang |
| Hap1 | PX622812 | *Haemaphysalis longicornis* | China-Shanxi-Wangjiazhuang |
| Hap1 | PX622813 | *Haemaphysalis longicornis* | China-Shanxi-Wangjiazhuang |
| Hap1 | PX622814 | *Haemaphysalis longicornis* | China-Shanxi-Wangjiazhuang |
| Hap1 | PX622815 | *Haemaphysalis longicornis* | China-Shanxi-Wangjiazhuang |
| Hap1 | PX622816 | *Haemaphysalis longicornis* | China-Shanxi-Wangjiazhuang |
| Hap1 | PX622817 | *Haemaphysalis longicornis* | China-Shanxi-Wangjiazhuang |
| Hap1 | PX622819 | *Haemaphysalis longicornis* | China-Shanxi-Wangjiazhuang |
| Hap1 | PX622820 | *Haemaphysalis longicornis* | China-Shanxi-Wangjiazhuang |
| Hap1 | PX622791 | *Haemaphysalis longicornis* | China-Shanxi-Baitupo |
| Hap1 | PX622793 | *Haemaphysalis longicornis* | China-Shanxi-Baitupo |
| Hap1 | PX622794 | *Haemaphysalis longicornis* | China-Shanxi-Baitupo |
| Hap1 | PX622795 | *Haemaphysalis longicornis* | China-Shanxi-Baitupo |
| Hap1 | PX622799 | *Haemaphysalis longicornis* | China-Shanxi-Baitupo |
| Hap1 | PX622800 | *Haemaphysalis longicornis* | China-Shanxi-Baitupo |
| Hap1 | PX622801 | *Haemaphysalis longicornis* | China-Shanxi-Baitupo |
| Hap1 | PX622802 | *Haemaphysalis longicornis* | China-Shanxi-Baitupo |
| Hap1 | PX622803 | *Haemaphysalis longicornis* | China-Shanxi-Baitupo |
| Hap1 | PX622805 | *Haemaphysalis longicornis* | China-Shanxi-Baitupo |
| Hap1 | PX622806 | *Haemaphysalis longicornis* | China-Shanxi-Baitupo |
| Hap1 | PX622807 | *Haemaphysalis longicornis* | China-Shanxi-Baitupo |
| Hap1 | PX622810 | *Haemaphysalis longicornis* | China-Shanxi-Baitupo |
| Hap1 | PX622826 | *Haemaphysalis longicornis* | China-Shanxi-Daxigou |
| Hap1 | PX622830 | *Haemaphysalis longicornis* | China-Shanxi-Daxigou |
| Hap1 | PX622767 | *Haemaphysalis longicornis* | China-Shanxi-Zuoquan |
| Hap1 | PX622770 | *Haemaphysalis longicornis* | China-Shanxi-Zuoquan |
| Hap1 | PX622771 | *Haemaphysalis longicornis* | China-Shanxi-Zuoquan |
| Hap1 | PX622772 | *Haemaphysalis longicornis* | China-Shanxi-Zuoquan |
| Hap1 | PX622777 | *Haemaphysalis longicornis* | China-Shanxi-Zuoquan |
| Hap1 | PX622778 | *Haemaphysalis longicornis* | China-Shanxi-Zuoquan |
| Hap1 | PX622779 | *Haemaphysalis longicornis* | China-Shanxi-Zuoquan |
| Hap1 | PX622784 | *Haemaphysalis longicornis* | China-Shanxi-Zuoquan |
| Hap1 | PX622788 | *Haemaphysalis longicornis* | China-Shanxi-Zuoquan |
| Hap1 | PX622789 | *Haemaphysalis longicornis* | China-Shanxi-Zuoquan |
| Hap1 | PX622722 | *Haemaphysalis longicornis* | China-Shanxi-Zhuanghe |
| Hap1 | PX622723 | *Haemaphysalis longicornis* | China-Shanxi-Zhuanghe |
| Hap1 | PX622724 | *Haemaphysalis longicornis* | China-Shanxi-Zhuanghe |
| Hap1 | PX622726 | *Haemaphysalis longicornis* | China-Shanxi-Zhuanghe |
| Hap1 | PX622748 | *Haemaphysalis longicornis* | China-Shanxi-Siyuan |
| Hap1 | PX622750 | *Haemaphysalis longicornis* | China-Shanxi-Siyuan |
| Hap1 | PX622751 | *Haemaphysalis longicornis* | China-Shanxi-Siyuan |
| Hap1 | PX622752 | *Haemaphysalis longicornis* | China-Shanxi-Siyuan |
| Hap1 | PX622753 | *Haemaphysalis longicornis* | China-Shanxi-Siyuan |
| Hap1 | PX622754 | *Haemaphysalis longicornis* | China-Shanxi-Siyuan |
| Hap1 | PX622756 | *Haemaphysalis longicornis* | China-Shanxi-Siyuan |
| Hap1 | PX622759 | *Haemaphysalis longicornis* | China-Shanxi-Siyuan |
| Hap1 | PX622760 | *Haemaphysalis longicornis* | China-Shanxi-Siyuan |
| Hap1 | PX622761 | *Haemaphysalis longicornis* | China-Shanxi-Siyuan |
| Hap1 | PX622762 | *Haemaphysalis longicornis* | China-Shanxi-Siyuan |
| Hap1 | PX622764 | *Haemaphysalis longicornis* | China-Shanxi-Siyuan |
| Hap1 | PX622741 | *Haemaphysalis longicornis* | China-Shanxi-Xiwangyong |
| Hap1 | PX622742 | *Haemaphysalis longicornis* | China-Shanxi-Xiwangyong |
| Hap1 | PX622743 | *Haemaphysalis longicornis* | China-Shanxi-Xiwangyong |
| Hap1 | PX622744 | *Haemaphysalis longicornis* | China-Shanxi-Xiwangyong |

Supplement Table S4 continued 1

| Haplotype | Acc. no | Host | Site |
| --- | --- | --- | --- |
| Hap1  Hap1 | PX622746  PX622747 | *Haemaphysalis longicornis*  *Haemaphysalis longicornis* | China-Shanxi-Xiwangyong  China-Shanxi-Xiwangyong |
| Hap1 | PX622765 | *Haemaphysalis longicornis* | China-Shanxi-Dongsitou |
| Hap1 | PX622738 | *Haemaphysalis longicornis* | China-Shanxi-Taling |
| Hap1 | PX622739 | *Haemaphysalis longicornis* | China-Shanxi-Taling |
| Hap1 | PX622735 | *Haemaphysalis longicornis* | China-Shanxi-Shipan |
| Hap1 | PX622736 | *Haemaphysalis longicornis* | China-Shanxi-Shipan |
| Hap1 | PX622737 | *Haemaphysalis longicornis* | China-Shanxi-Shipan |
| Hap1 | PX622738 | *Haemaphysalis longicornis* | China-Shanxi-Shipan |
| Hap1 | PX622739 | *Haemaphysalis longicornis* | China-Shanxi-Shipan |
| Hap1 | PX622740 | *Haemaphysalis longicornis* | China-Shanxi-Houbu |
| Hap1 | OR797028.1 | Tick | China-Anhui |
| Hap1 | KU723457.1 | *Rhipicephalus sanguineus* | China-Xinjiang |
| Hap1 | LC457964.1 | *Haemaphysalis flava* | Japan |
| Hap2 | PX622822 | *Haemaphysalis longicornis* | China-Shanxi-Wangjiazhuang |
| Hap2 | MN795156.1 | Cattle | Iran |
| Hap3 | PX622818 | *Haemaphysalis longicornis* | China-Shanxi-Wangjiazhuang |
| Hap3 | PX622821 | *Haemaphysalis longicornis* | China-Shanxi-Wangjiazhuang |
| Hap3 | PX622824 | *Haemaphysalis longicornis* | China-Shanxi-Wangjiazhuang |
| Hap3 | PX622792 | *Haemaphysalis longicornis* | China-Shanxi-Baitupo |
| Hap3 | PX622798 | *Haemaphysalis longicornis* | China-Shanxi-Baitupo |
| Hap3 | PX622809 | *Haemaphysalis longicornis* | China-Shanxi-Baitupo |
| Hap3 | PX622827 | *Haemaphysalis longicornis* | China-Shanxi-Daxigou |
| Hap3 | PX622828 | *Haemaphysalis longicornis* | China-Shanxi-Daxigou |
| Hap3 | PX622829 | *Haemaphysalis longicornis* | China-Shanxi-Daxigou |
| Hap3 | PX622768 | *Haemaphysalis longicornis* | China-Shanxi-Zuoquan |
| Hap3 | PX622774 | *Haemaphysalis longicornis* | China-Shanxi-Zuoquan |
| Hap3 | PX622755 | *Haemaphysalis longicornis* | China-Shanxi-Siyuan |
| Hap3 | PX622757 | *Haemaphysalis longicornis* | China-Shanxi-Siyuan |
| Hap3 | PX622745 | *Haemaphysalis longicornis* | China-Shanxi-Xiwangyong |
| Hap3 | DQ449947.1 | *Ixodes persulcatus* | China-Beijing |
| Hap3 | DQ449948.1 | *Dermacentor silvarum* | China-Jilin |
| Hap3 | OQ701070.1 | *Haemaphysalis longicornis* | China-Hebei |
| Hap3 | JN990106.1 | Tick | China-JiangSu |
| Hap3 | KP062963.1 | Goat | China-Zhejiang |
| Hap3 | KP062962.1 | Goat | China-Zhejiang |
| Hap3 | KY242452.1 | *Haemaphysalis longicornis* | China-Shandong |
| Hap3 | KT951192.1 | Tick | China-Gansu |
| Hap3 | PP905412.1 | *Ixodes scapularis* | USA |
| Hap3 | PQ045907.1 | *Ixodes scapularis* | USA |
| Hap3 | MW899039.1 | *Ixodes scapularis* | USA |
| Hap3 | MW899038.1 | *Ixodes scapularis* | USA |
| Hap3 | MW899038.1 | *Ixodes scapularis* | USA |
| Hap3 | KM215233.1 | *Ixodes ricinus* | Slovenia |
| Hap3 | KM215227.1 | *Ixodes ricinus* | Slovenia |
| Hap3 | KJ542649.1 | *Ixodes ricinus* | Slovenia |
| Hap3 | JX627378.1 | *Ixodes ricinus* | Germany |
| Hap3 | JX627376.1 | *Ixodes ricinus* | Germany |
| Hap3 | JX627374.1 | *Ixodes ricinus* | Germany |
| Hap3 | JN244016.1 | *Capreolus capreolus* | Germany |
| Hap3 | MZ348265.1 | *Ixodes ricinus* | Germany |
| Hap3 | KC833753.1 | *Ixodes ricinus* | Germany |
| Hap3 | JX627370.1 | *Ixodes ricinus* | Germany |
| Hap3 | FJ788513.1 | *Ixodes ricinus* | Germany |
| Hap3 | JX627372.1 | *Ixodes ricinus* | Germany |
| Hap3 | JX909360.1 | *Ixodes ricinus* | Germany |

Supplement Table S4 continued 2

| Haplotype | Acc. no | Host | Site |
| --- | --- | --- | --- |
| Hap3 | JF895448.1 | Ixodes ricinus | Germany |
| Hap3 | JQ026311.1 | Bos taurus | Germany |
| Hap3 | JN244015.1 | Capreolus capreolus | Germany |
| Hap3 | KC833754.1 | Wild Boar | Germany |
| Hap3 | JX627367.1 | Deer | Germany |
| Hap3 | JX627364.1 | Deer | Germany |
| Hap3 | KU587123.1 | *Ixodes ricinus* | Germany |
| Hap3 | MN252874.1 | Avian | Greece |
| Hap3 | OQ727068.1 | Tick | Malawi |
| Hap3 | OQ727069.1 | Tick | Malawi |
| Hap3 | OP824620.1 | Cattle | Malawi |
| Hap3 | OP824619.1 | Cattle | Malawi |
| Hap3 | MW922756.1 | *Ixodes ricinus* | Estonia |
| Hap3 | HQ629925.1 | *Ixodes ricinus* | Estonia |
| Hap3 | HQ629923.1 | *Ixodes ricinus* | Estonia |
| Hap3 | MH256660.1 | *Ixodes ricinus* | Finland |
| Hap3 | PV186471.1 | *Haemaphysalis intermedia* | India |
| Hap3 | MG050135.1 | *Rhipicephalus sanguineus* | India |
| Hap3 | MG050134.1 | *Rhipicephalus sanguineus* | India |
| Hap3 | MG050132.1 | *Rhipicephalus sanguineus* | India |
| Hap3 | OR268762.1 | *Ixodes ricinus* | United-Kingdom |
| Hap3 | AY149637.1 | *Ixodes ricinus* | United-Kingdom |
| Hap3 | AY149636.1 | *Ixodes ricinus* | United-Kingdom |
| Hap3 | AY082656.1 | *Clethrionomys glareolus* | United-Kingdom |
| Hap3 | PP663805.1 | *Ixodes nipponensis* | South Korea |
| Hap3 | PP663794.1 | *Ixodes nipponensis* | South Korea |
| Hap3 | MW715067.1 | Horse | South Korea |
| Hap3 | MW715064.1 | Horse | South Korea |
| Hap3 | PV577431.1 | *Haemaphysalis sp.* | South Korea |
| Hap3 | PP346261.1 | *Hydropotes inermis argyropus* | South Korea |
| Hap3 | OQ552617.1 | *Ixodes nipponensis* | South Korea |
| Hap3 | MK283927.1 | Tick | South Korea |
| Hap3 | JQ086317.1 | Tick | South Korea |
| Hap3 | OQ518411.1 | Dog | South Korea |
| Hap3 | MK239931.1 | Dog | South Korea |
| Hap3 | MF787270.1 | *Bos taurus* | South Korea |
| Hap3 | OR865211.1 | *Haemaphysalis longicornis* | South Korea |
| Hap3 | PP663780.1 | *Haemaphysalis longicornis* | South Korea |
| Hap3 | KX646193.1 | *Ixodes ricinus* | Poland |
| Hap3 | KP245908.1 | *Ixodes ricinus* | Poland |
| Hap3 | KP245906.1 | *Ixodes ricinus* | Poland |
| Hap3 | MW879362.1 | Deer | Poland |
| Hap3 | OP683230.1 | *Dermacentor reticulatus* | Lithuania |
| Hap3 | OP683228.1 | *Dermacentor reticulatus* | Lithuania |
| Hap3 | JN181081.1 | *Ixodes ricinus* | Lithuania |
| Hap3 | JN181079.1 | *Ixodes ricinus* | Lithuania |
| Hap3 | ON614171.1 | Tick | Serbia |
| Hap3 | MW900166.1 | *Ixodes ricinus* | Serbia |
| Hap3 | PP763384.1 | *Hyalomma aegyptium* | Iran |
| Hap3 | LC457961.1 | *Haemaphysalis longicornis* | Japan |
| Hap3 | KY319199.1 | *Ixodes ricinus* | Italy |
| Hap3 | KY319196.1 | *Ixodes ricinus* | Italy |
| Hap3 | KY319192.1 | *Ixodes ricinus* | Italy |
| Hap3 | KY319191.1 | *Ixodes ricinus* | Italy |
| Hap3 | KP877313.1 | Tick | Italy |

Supplement Table S4 continued 3

| Haplotype | Acc. no | Host | Site |
| --- | --- | --- | --- |
| Hap3 | PQ657431.1 | Homo sapiens | Italy |
| Hap3 | KF034787.1 | *Haemaphysalis sulcata* | Turkey |
| Hap3 | MN611757.1 | Sheep | Turkey |
| Hap3 | JN181074.1 | *Ixodes ricinus* | Norway |
| Hap3 | JN181072.1 | *Ixodes ricinus* | Norway |
| Hap3 | MT221234.1 | *Alces alces* | Norway |
| Hap3 | MT221233.1 | *Alces alces* | Norway |
| Hap3 | MH487660.1 | Deer | Mexico |
| Hap3 | GU111747.1 | *Ixodes ricinus* | Spain |
| Hap3 | GU111746.1 | *Ixodes ricinus* | Spain |
| Hap3 | AY587607.1 | *Ixodes persulcatus* | Russia |
| Hap3 | KC753763.1 | *Ixodes persulcatus* | Russia |
| Hap3 | KU534875.1 | *Rhipicephalus sanguineus* | Costa_Rica |
| Hap3 | KU534874.1 | *Rhipicephalus sanguineus* | Costa_Rica |
| Hap3 | FJ538291.1 | *Bos taurus* | Switzerland |
| Hap3 | OR426543.1 | *Ixodes ricinus* | France |
| Hap3 | OR426542.1 | *Ixodes ricinus* | France |
| Hap4 | PX622823 | *Haemaphysalis longicornis* | China-Shanxi-Wangjiazhuang |
| Hap4 | PX622796 | *Haemaphysalis longicornis* | China-Shanxi-Baitupo |
| Hap4 | PX622797 | *Haemaphysalis longicornis* | China-Shanxi-Baitupo |
| Hap4 | PX622831 | *Haemaphysalis longicornis* | China-Shanxi-Daxigou |
| Hap4 | PX622773 | *Haemaphysalis longicornis* | China-Shanxi-Zuoquan |
| Hap4 | PX622776 | *Haemaphysalis longicornis* | China-Shanxi-Zuoquan |
| Hap4 | PX622780 | *Haemaphysalis longicornis* | China-Shanxi-Zuoquan |
| Hap4 | PX622781 | *Haemaphysalis longicornis* | China-Shanxi-Zuoquan |
| Hap4 | PX622783 | *Haemaphysalis longicornis* | China-Shanxi-Zuoquan |
| Hap4 | PX622785 | *Haemaphysalis longicornis* | China-Shanxi-Zuoquan |
| Hap4 | PX622786 | *Haemaphysalis longicornis* | China-Shanxi-Zuoquan |
| Hap4 | PX622787 | *Haemaphysalis longicornis* | China-Shanxi-Zuoquan |
| Hap4 | PX622790 | *Haemaphysalis longicornis* | China-Shanxi-Zuoquan |
| Hap5 | PX622825 | *Haemaphysalis longicornis* | China-Shanxi-Wangjiazhuang |
| Hap5 | PX622808 | *Haemaphysalis longicornis* | China-Shanxi-Baitupo |
| Hap5 | PX622769 | *Haemaphysalis longicornis* | China-Shanxi-Zuoquan |
| Hap5 | PX622728 | *Haemaphysalis longicornis* | China-Shanxi-Zhuanghe |
| Hap5 | PX622758 | *Haemaphysalis longicornis* | China-Shanxi-Siyuan |
| Hap5 | PX622732 | *Haemaphysalis longicornis* | China-Shanxi-Taling |
| Hap5 | PX622733 | *Haemaphysalis longicornis* | China-Shanxi-Taling |
| Hap5 | PX622734 | *Haemaphysalis longicornis* | China-Shanxi-Taling |
| Hap5 | KJ459350.1 | Tick | China-Xinjiang |
| Hap6 | PX622804 | *Haemaphysalis longicornis* | China-Shanxi-Baitupo |
| Hap6 | PX622749 | *Haemaphysalis longicornis* | China-Shanxi-Siyuan |
| Hap7 | PX622766 | *Haemaphysalis longicornis* | China-Shanxi-Zuoquan |
| Hap8 | PX622775 | *Haemaphysalis longicornis* | China-Shanxi-Zuoquan |
| Hap8 | PP663794.1 | *Ixodes nipponensis* | South Korea |
| Hap8 | PV577435.1 | *Haemaphysalis sp.* | South Korea |
| Hap8 | MN559940.1 | *Hydropotes inermis* | South Korea |
| Hap8 | MH338210.1 | Deer | South Korea |
| Hap8 | MT126499.1 | Horse | Japan |
| Hap8 | MT126498.1 | Horse | Japan |
| Hap8 | PP346264.1 | Goat | Viet Nam |
| Hap8 | PP346263.1 | Cattle | Philippines |
| Hap9 | PX622782 | *Haemaphysalis longicornis* | China-Shanxi-Zuoquan |
| Hap9 | OQ308965.1 | *Haemaphysalis leachi* | Kenya |
| Hap10 | PX622725 | *Haemaphysalis longicornis* | China-Shanxi-Zhuanghe |
| Hap11 | PX622727 | *Haemaphysalis longicornis* | China-Shanxi-Zhuanghe |

Supplement Table S4 continued 4

| Haplotype | Acc. no | Host | Site |
| --- | --- | --- | --- |
| Hap12 | PX622729 | *Haemaphysalis longicornis* | China-Shanxi-Zhuanghe |
| Hap13 | PX622763 | *Haemaphysalis longicornis* | China-Shanxi-Siyuan |
| Hap14 | AF530575.1 | Tick | USA |
| Hap15 | MW422836.1 | Tick | Iraq |
| Hap15 | KU723458.1 | Hyalomma asiaticum | Kazakhstan |
| Hap15 | KM009069.1 | Rhipicephalus microplus | Colombia |
| Hap15 | PV202459.1 | Tick | China-Hubei |
| Hap16 | MW922754.1 | *Ixodes ricinus* | Estonia |
| Hap16 | KX646191.1 | *Ixodes ricinus* | Poland |
| Hap16 | OR532503.1 | *Ixodes ricinus* | Poland |
| Hap16 | PV124223.1 | *Ixodes ricinus* | Poland |
| Hap16 | JX298075.1 | *Ixodes ricinus* | Ukraine |
| Hap16 | HQ629918.1 | *Ixodes ricinus* | Russia |
| Hap16 | HQ629911.1 | *Ixodes ricinus* | Russia |
| Hap16 | HQ629921.1 | *Ixodes ricinus* | Belarus |
| Hap16 | HQ629916.1 | *Ixodes ricinus* | Belarus |
| Hap16 | FJ538290.1 | *Bos taurus* | Switzerland |
| Hap16 | EF217403.1 | *Dama dama* | Czech Republic |
| Hap16 | EF217400.1 | *Dama dama* | Czech Republic |
| Hap17 | HQ629922.1 | *Ixodes ricinus* | Estonia |
| Hap18 | MK757727.1 | *Rhipicephalus sanguineus* | India |
| Hap19 | MK757729.1 | *Rhipicephalus sanguineus* | India |
| Hap20 | DQ648489.1 | Cattle | India |
| Hap20 | PV386796.1 | Cattle | India |
| Hap20 | PV386795.1 | Cattle | India |
| Hap21 | OP683233.1 | *Ixodes ricinus* | Poland |
| Hap22 | JN107802.1 | Homo sapiens | Poland |
| Hap23 | MN795157.1 | Cattle | Iran |
| Hap24 | MN795151.1 | Cattle | Iran |
| Hap25 | MN795153.1 | Cattle | Iran |
| Hap26 | LC457963.1 | *Haemaphysalis longicornis* | Japan |
| Hap27 | JQ685510.1 | *Haemaphysalis douglasi* | Japan |
| Hap28 | PQ657429.1 | Homo sapiens | Italy |
| Hap29 | PP763388.1 | *Hyalomma aegyptium* | Turkey |
| Hap30 | PP763386.1 | *Hyalomma aegyptium* | Turkey |
| Hap31 | MW800890.1 | *Ixodes ricinus* | Spain |
| Hap32 | MW800888.1 | *Ixodes ricinus* | Spain |
| Hap33 | OR623250.1 | *Ixodes ricinus* | Spain |
| Hap34 | KU723456.1 | *Hyalomma asiaticum* | China-Xinjiang |
| Hap35 | JX914659.1 | Tick | China-Gansu |

Supplement Table S5 Haplotypes of *Anaplasma ovis* involved in figure 4

| Haplotype | Acc. no | Host | Site |
| --- | --- | --- | --- |
| Hap1 | PX622859 | *Haemaphysalis longicornis* | China-shanxi-Wangjiazhuang |
| Hap2 | PX622860 | *Haemaphysalis longicornis* | China-shanxi-Wangjiazhuang |
| Hap2 | PX622865 | *Haemaphysalis longicornis* | China-shanxi-Wangjiazhuang |
| Hap2 | PX622866 | *Haemaphysalis longicornis* | China-shanxi-Wangjiazhuang |
| Hap2 | PX622837 | *Haemaphysalis longicornis* | China-shanxi-Baitupo |
| Hap2 | PX622838 | *Haemaphysalis longicornis* | China-shanxi-Baitupo |
| Hap2 | PX622839 | *Haemaphysalis longicornis* | China-shanxi-Baitupo |
| Hap2 | PX622842 | *Haemaphysalis longicornis* | China-shanxi-Baitupo |
| Hap2 | PX622844 | *Haemaphysalis longicornis* | China-shanxi-Baitupo |
| Hap2 | PX622845 | *Haemaphysalis longicornis* | China-shanxi-Baitupo |
| Hap2 | PX622846 | *Haemaphysalis longicornis* | China-shanxi-Baitupo |
| Hap2 | PX622847 | *Haemaphysalis longicornis* | China-shanxi-Baitupo |
| Hap2 | PX622848 | *Haemaphysalis longicornis* | China-shanxi-Baitupo |
| Hap2 | PX622849 | *Haemaphysalis longicornis* | China-shanxi-Baitupo |
| Hap2 | PX622851 | *Haemaphysalis longicornis* | China-shanxi-Baitupo |
| Hap2 | PX622852 | *Haemaphysalis longicornis* | China-shanxi-Baitupo |
| Hap2 | PX622853 | *Haemaphysalis longicornis* | China-shanxi-Baitupo |
| Hap2 | PX622854 | *Haemaphysalis longicornis* | China-shanxi-Baitupo |
| Hap2 | PX622855 | *Haemaphysalis longicornis* | China-shanxi-Baitupo |
| Hap2 | PX622856 | *Haemaphysalis longicornis* | China-shanxi-Baitupo |
| Hap2 | PX622857 | *Haemaphysalis longicornis* | China-shanxi-Baitupo |
| Hap2 | PP106358.1 | *Dermacentor silvarum* | China-Beijing |
| Hap2 | OP363198.1 | *Ixodes persulcatus* | China-Hubei |
| Hap2 | PV202458.1 | Tick | China-Hubei |
| Hap2 | PV202457.1 | Tick | China-Hubei |
| Hap2 | PV202454.1 | Tick | China-Hubei |
| Hap2 | PV202452.1 | Tick | China-Hubei |
| Hap2 | MT279318.1 | Sheep | China-XinJiang |
| Hap2 | MT279317.1 | Sheep | China-XinJiang |
| Hap2 | MT279315.1 | Sheep | China-XinJiang |
| Hap2 | MT279314.1 | Sheep | China-XinJiang |
| Hap2 | MW600412.1 | Sheep | China-XinJiang |
| Hap2 | OR214929.1 | Tick | China-Qinghai |
| Hap2 | OR214928.1 | Tick | China-Qinghai |
| Hap2 | OQ701064.1 | *Haemaphysalis longicornis* | China-Hebei |
| Hap2 | OQ701063.1 | *Haemaphysalis longicornis* | China-Hebei |
| Hap2 | OQ701062.1 | *Haemaphysalis longicornis* | China-Hebei |
| Hap2 | KJ659039.1 | Deer | China-Gansu |
| Hap2 | KJ659038.1 | Deer | China-Gansu |
| Hap2 | KJ639881.1 | Deer | China-Gansu |
| Hap2 | KJ639879.1 | Deer | China-Gansu |
| Hap2 | KU569700.1 | Sheep | Kenya |
| Hap2 | OR150331.1 | *Rhipicephalus turanicus* | Kyrgyzstan |
| Hap2 | OR150329.1 | *Dermacentor sp.* | Kyrgyzstan |
| Hap2 | OR150327.1 | *Hyalomma marginatum* | Kyrgyzstan |
| Hap2 | JF514511.1 | Goat | Iran |
| Hap2 | JF514503.1 | Sheep | Iran |
| Hap2 | KM517582.1 | Tick | Iran |
| Hap2 | KM517581.1 | Tick | Iran |
| Hap2 | KM517580.1 | Tick | Iran |
| Hap2 | MK806691.1 | *Rhipicephalus sanguineus* | Iran |
| Hap2 | KF034788.1 | *Rhipicephalus bursa* | Turkey |
| Hap2 | KF414717.1 | *Rhipicephalus evertsi* | South Africa |
| Hap2 | GQ857077.1 | Sheep | Italy |
| Hap3 | PX622861 | *Haemaphysalis longicornis* | China-shanxi-Wangjiazhuang |
| Hap3 | PX622863 | *Haemaphysalis longicornis* | China-shanxi-Wangjiazhuang |

Supplement Table S5 continued

| Haplotype | Acc. no | Host | Site |
| --- | --- | --- | --- |
| Hap3 | PX622850 | *Haemaphysalis longicornis* | China-shanxi-Baitupo |
| Hap3 | PX622858 | *Haemaphysalis longicornis* | China-shanxi-Baitupo |
| Hap4 | PX622862 | *Haemaphysalis longicornis* | China-shanxi-Wangjiazhuang |
| Hap5 | PX622864 | *Haemaphysalis longicornis* | China-shanxi-Wangjiazhuang |
| Hap6 | PX622840 | *Haemaphysalis longicornis* | China-shanxi-Baitupo |
| Hap6 | PX622841 | *Haemaphysalis longicornis* | China-shanxi-Baitupo |
| Hap7 | PX622843 | *Haemaphysalis longicornis* | China-shanxi-Baitupo |
| Hap7 | PX622832 | *Haemaphysalis longicornis* | China-shanxi-Zhuanghe |
| Hap7 | PP106360.1 | *Dermacentor silvarum* | China-Beijing |
| Hap7 | PP106354.1 | *Dermacentor silvarum* | China-Beijing |
| Hap7 | JN187093.1 | *Haemaphysalis longicorni* | China-Xinjiang |
| Hap7 | OR214932.1 | Tick | China-Qinghai |
| Hap7 | MN795144.1 | Sheep | Iran |
| Hap7 | KC484562.1 | *Dermacentor* | Russia |
| Hap8 | PX622867 | *Haemaphysalis longicornis* | China-shanxi-Daxigou |
| Hap9 | PX622833 | *Haemaphysalis longicornis* | China-shanxi-Zhuanghe |
| Hap9 | PX622836 | *Haemaphysalis longicornis* | China-shanxi-Shipan |
| Hap9 | PX622834 | *Haemaphysalis longicornis* | China-shanxi-Houbu |
| Hap10 | PX622835 | *Haemaphysalis longicornis* | China-shanxi-Xiwangyong |
| Hap11 | MT279319.1 | Sheep | China-XinJiang |
| Hap12 | KU569702.1 | Sheep | Kenya |
| Hap13 | KU569701.1 | *Nanger granti* | Kenya |
| Hap14 | PP961318.1 | Sheep | Iraq |
| Hap15 | PP961317.1 | Sheep | Iraq |
| Hap16 | PP961312.1 | Sheep | Iraq |
| Hap17 | MN795156.1 | Cattle | Iran |
| Hap18 | MN795155.1 | Cattle | Iran |
| Hap19 | MN795152.1 | Cattle | Iran |
| Hap20 | MN795149.1 | Goat | Iran |
| Hap21 | MN795147.1 | Sheep | Iran |
| Hap22 | MN795146.1 | Sheep | Iran |
| Hap23 | MN795143.1 | Sheep | Iran |
| Hap24 | GQ857075.1 | Sheep | Italy |
| Hap25 | MZ348265.1 | *Ixodes ricinus* | Germany |
| Hap25 | MZ348262.1 | *Ixodes ricinus* | Germany |
| Hap25 | MZ348259.1 | *Ixodes ricinus* | Germany |
| Hap25 | MZ348256.1 | *Ixodes ricinus* | Germany |

Supplement Table S6 Haplotypes of *Anaplasma marginale* involved in figure 5

| Haplotype | Acc. no | Host | Site |
| --- | --- | --- | --- |
| Hap1 | PX622868 | *Haemaphysalis longicornis* | China-Shanxi-Siyuan |
| Hap1 | MW412723.1 | *Rhipicephalus microplus* | China-Sichuan |
| Hap1 | HM439433.1 | Cattle | China-Zhejiang |
| Hap1 | OR520946.1 | Tick | China-Beijing |
| Hap1 | PQ892078.1 | *Rhipicephalus microplus* | China-Jiangxi |
| Hap1 | OR835876.1 | *Rhipicephalus microplus* | China-Hainan |
| Hap1 | OL660546.1 | Bos taurus | China-Taiwan |
| Hap1 | AF309868.1 | - | USA |
| Hap1 | PV569600.1 | Cattle | Turkey |
| Hap1 | KJ183083.1 | Cattle | Turkey |
| Hap1 | OP379590.1 | *Rhipicephalus microplus* | Thailand |
| Hap1 | OP379612.1 | *Rhipicephalus microplus* | Thailand |
| Hap1 | MK310488.1 | *Hyalomma schulzei* | Iran |
| Hap1 | MH686047.1 | Cattle | Viet_Nam |
| Hap1 | JQ735904.1 | Cattle | Mongolia |
| Hap1 | JQ839012.1 | *Boophilus microplus* | Philippines |
| Hap1 | JQ839009.1 | *Boophilus microplus* | Philippines |
| Hap1 | AF414871.1 | Black wildebeest | South Africa |
| Hap1 | FJ155998.1 | *Oryx gazella gazella* | South Africa |
| Hap1 | PV767334.1 | Cattle | Egypt |
| Hap1 | PX242208.1 | Cattle | Egypt |
| Hap1 | MN726536.1 | Tick | Egypt |
| Hap1 | OR724728.1 | *Rhipicephalus microplus* | Panama |
| Hap1 | PQ865287.1 | *Rhipicephalus microplus* | Malaysia |
| Hap1 | PX446450.1 | Cattle | Pakistan |
| Hap1 | OL409039.1 | Goat | Pakistan |
| Hap1 | OQ976985.1 | Cattle | Pakistan |
| Hap1 | OQ619390.1 | Tick | Cuba |
| Hap1 | PV290698.1 | Cattle | India |
| Hap1 | KF696858.1 | Cattle | India |
| Hap1 | OP851751.1 | Bovine | India |
| Hap1 | JF949768.1 | *Rhipicephalus decoloratus* | Nigeria |
| Hap1 | KJ095114.1 | Cattle | Nigeria |
| Hap1 | OP353615.1 | Bovine | Trinidad and Tobago |
| Hap1 | PP933767.1 | *Canis lupus* | Trinidad and Tobago |
| Hap1 | PP933762.1 | Bos taurus | Trinidad and Tobago |
| Hap1 | MZ798902.1 | Bos taurus | Mexico |
| Hap1 | KM009068.1 | *Rhipicephalus microplus* | Colombia |
| Hap2 | HM538193.1 | Buffalo | China-Yunnan |
| Hap2 | MG018437.1 | *Rhipicephalus microplus* | India |
| Hap3 | AJ633048.1 | Cattle | China-Henan |
| Hap4 | OP353616.1 | Bovine | Trinidad and Tobago |
| Hap5 | KP877313.1 | Tick | Italy |
| Hap6 | PV052636.1 | *Amblyomma mixtum* | Mexico |

Supplement Table S7 Haplotypes of *Anaplasma bovis* involved in figure 6

| Haplotype | Acc. no | Host | Site |
| --- | --- | --- | --- |
| Hap1 | PX622869 | *Haemaphysalis longicornis* | China-Shanxi-Houbu |
| Hap2 | MZ231112.1 | Yak | China-Qinghai |
| Hap2 | OQ701067.1 | *Haemaphysalis longicornis* | China-Hebei |
| Hap2 | KP314239.1 | Tick | China-Shandong |
| Hap2 | KJ659040.1 | Deer | China-Gansu |
| Hap2 | MK271373.1 | Goat | China-Gansu |
| Hap2 | KY655799.1 | Goat | Tunisia |
| Hap2 | MF000920.1 | cattle | South Korea |
| Hap2 | OP535549.1 | *Haemaphysalis longicornis* | South Korea |
| Hap2 | GU556627.1 | *Hydropotes inermis argyropus* | South Korea |
| Hap2 | AF470698.1 | *Haemaphysalis longicornis* | South Korea |
| Hap2 | PP663807.1 | *Haemaphysalis longicornis* | South Korea |
| Hap2 | MH338212.1 | Deer | South Korea |
| Hap2 | OQ552618.1 | *Haemaphysalis longicornis* | South Korea |
| Hap2 | MH794247.1 | Horse | South Korea |
| Hap2 | JQ086318.1 | Tick | South Korea |
| Hap2 | MT036513.1 | Sheep | Russia |
| Hap2 | PQ305632.1 | *Haemaphysalis punctata* | Kyrgyzstan |
| Hap2 | MN213735.1 | Giraffe | Pakistan |
| Hap2 | PP348760.1 | Goat | Indonesia |
| Hap2 | KU242422.1 | Sheep | Iran |
| Hap2 | KU569703.1 | *Nanger granti* | Kenya |
| Hap2 | KJ183084.1 | Cattle | Turkey |
| Hap3 | OQ826693.1 | Cattle | China-Qinghai |
| Hap4 | OK560164.1 | *Bandicota indica* | China-Taiwan |
| Hap5 | MN044717.1 | *Rhipicephalus microplus* | China-Shaanxi |
| Hap5 | MH255941.1 | Cattle | China-Shaanxi |
| Hap5 | MH255936.1 | Cattle | China-Shaanxi |
| Hap5 | MH255925.1 | Goat | China-Shaanxi |
| Hap5 | KY242455.1 | *Haemaphysalis longicornis* | China-Shandong |
| Hap5 | MF289479.1 | Cattle | China-Chongqing |
| Hap5 | KJ639883.1 | Deer | China-Gansu |
| Hap5 | MH180816.1 | Himalayan goral | China-Gansu |
| Hap5 | KX450273.1 | Tick | China-Henan |
| Hap5 | MZ230569.1 | *Haemaphysalis longicornis* | China-Henan |
| Hap5 | PP087918.1 | Tick | China-Changsha |
| Hap5 | MH085195.1 | Deer | China-Heilongjiang |
| Hap5 | PV716336.1 | - | Pakistan |
| Hap5 | OQ533602.1 | Tick | Pakistan |
| Hap5 | OR823814.1 | Tick | Malawi |
| Hap5 | OP824766.1 | Cattle | Malawi |
| Hap5 | PP265063.1 | *Haemaphysalis bispinosa* | Bangladesh |
| Hap5 | MG700528.1 | Cattle | Tanzania |
| Hap6 | PQ206196.1 | Tick | China-Chongqing |
| Hap6 | KY548389.1 | Dog | South Korea |
| Hap6 | HM131217.1 | Dog | Japan |
| Hap6 | GU937012.1 | Raccoon | Japan |
| Hap6 | AB723714.1 | *Prionailurus bengalensis euptilurus* | Japan |
| Hap6 | JX082006.1 | *Haemaphysalis megaspinosa* | Japan |
| Hap7 | MK345480.1 | Cattle | China-Xinjiang |
| Hap8 | OR797025.1 | Tick | China-Anhui |
| Hap9 | KY425445.1 | *Amblyomma triguttatum* | Australia |
| Hap10 | MH244925.1 | Cattle | India |
| Hap10 | MH379982.1 | Cattle | India |
| Hap11 | MG018451.1 | *Haemaphysalis bispinosa* | India |

Supplement Table S7 continued

| Haplotype | Acc. no | Host | Site |
| --- | --- | --- | --- |
| Hap12 | MT800793.1 | Horse | South Korea |
| Hap13 | KM114611.1 | *Macaca fascicularis* | Malaysia |
| Hap14 | AB983438.1 | *Prionailurus bengalensis euptilurus* | Japan |
| Hap15 | LC457964.1 | *Haemaphysalis flava* | Japan |
| Hap16 | OR234597.1 | *Haemaphysalis punctata* | Kyrgyzstan |
| Hap17 | JF952893.1 | *Blastoceros dichotomus* | Brazil |
| Hap18 | KP659195.1 | *Haemaphysalis shimoga* | Thailand |
| Hap19 | KF414716.1 | *Rhipicephalus evertsi evertsi* | South Africa |
